# Hesperidin Displays Relevant Role in the Nutrigenomic Effect of Orange Juice on Blood Leukocytes in Human Volunteers: A Randomized Controlled Cross-Over Study

**DOI:** 10.1371/journal.pone.0026669

**Published:** 2011-11-16

**Authors:** Dragan Milenkovic, Christiane Deval, Claude Dubray, Andrzej Mazur, Christine Morand

**Affiliations:** 1 INRA, UMR 1019, UNH, CRNH Auvergne, Clermont-Ferrand, Clermont Université, Université d'Auvergne, Unité de Nutrition Humaine, BP 10448, Clermont-Ferrand, France; 2 Inserm, CIC 501, Clermont-Ferrand, France; Institut Pluridisciplinaire Hubert Curien, France

## Abstract

**Background:**

We previously showed, in healthy, middle-aged, moderately overweight men, that orange juice decreases diastolic blood pressure and significantly improves postprandial microvascular endothelial reactivity and that hesperidin could be causally linked to the observed beneficial effect of orange juice. The objective was to determine the effect of chronic consumption of orange juice on the gene expression profile of leukocytes in healthy volunteers and to assess to what extent hesperidin is involved in the effect of orange juice.

**Methodology/Principal Findings:**

Volunteers were included in a randomized, controlled, crossover study. Throughout three 4-week periods, volunteers consumed daily: 500 ml orange juice, 500 ml control drink plus hesperidin or 500 ml control drink and placebo. Blood samplings were performed on 10 overnight-fasted subjects after the 4-week treatment period. Global gene expression profiles were determined using human whole genome cDNA microarrays. Both orange juice and hesperidin consumption significantly affected leukocyte gene expression. Orange juice consumption induced changes in expression of, 3,422 genes, while hesperidin intake modulated the expression of 1,819 genes. Between the orange juice and hesperidin consumption groups, 1,582 regulated genes were in common. Many of these genes are implicated in chemotaxis, adhesion, infiltration and lipid transport, which is suggestive of lower recruitment and infiltration of circulating cells to vascular wall and lower lipid accumulation.

**Conclusions:**

This study shows that regular consumption of orange juice for 4 weeks alters leukocyte gene expression to an anti-inflammatory and anti-atherogenic profile, and hesperidin displays a relevant role in the genomic effect of this beverage.

**Trial Registration:**

ClinicalTrials.gov NCT 00983086

## Introduction

We previously showed, in healthy, middle-aged, but moderately overweight men, that orange juice decreased diastolic blood pressure and significantly improved postprandial microvascular endothelial reactivity [1]. Furthermore, our study strongly suggested that hesperidin, its major flavonoid, is causally linked to the observed beneficial effect of orange juice. These vascular protective effects of orange juice and the possible specific role of hesperidin in mediating these effects are in agreement with a recent prospective study that showed convincing results for an association between the dietary intake of flavanones and flavanone-rich foods and reduced risks of coronary heart diseases [2]. Other clinical studies in healthy subjects have also shown that orange juice consumption reduced oxidative DNA damage and may prevent meal-induced oxidative and inflammatory stress in circulating blood mononuclear cells [3], [4]. The reduction of reactive oxygen species generation and NF-κB binding following orange juice intake could possibly be due to its flavonoid content, as suggested by in vitro results. In fact, these changes occurred when mononuclear cells were incubated with hesperetin, while fructose or ascorbic acid did not cause any change [3]. Nevertheless, much remains to be done to advance the understanding of the mechanisms by which the orange juice and some of its constituents could exert protective health effects. Nevertheless, much work remains in order to advance our understanding of the mechanisms by which orange juice and some of its constituents could exert protective health effects.

For some foods rich in flavonoids, such as tea or cocoa, the role of these bioactive compounds in vascular protection has been demonstrated in clinical trials [5], [6], studies on animal models of atherosclerosis and in *in vitro* studies using vascular cells [7], [8]. Some of these studies have suggested that flavonoids could mediate vascular cell function through the modulation of gene expression and intracellular signaling pathways [9]–[11]. In addition, recent findings from animal studies suggest that the actions of flavonoids are related to their capacity to interact with the cellular signaling cascades that regulate transcription factors and as a consequence, expression of genes and proteins [12], [13]. More recently, transcriptome analysis of aortas from mice fed naringin revealed that the anti-atherogenic effect of this grapefruit flavonoid might be linked to changes in gene expression that play a role in the preservation of the vascular wall [14].

Few human studies have shown the potential use of gene expression profiling in blood leukocytes to study the effects of diet on gene expression modulation. It has been proposed that modulation of gene expression in these cells might be related to the various clinical and biochemical changes that occur during cardiovascular disease (CVD) development [15]–[17]. Nevertheless, the nutrigenomic impact of dietary flavonoids has not been extensively investigated. To our knowledge, only one recent study has examined the effect of quercetin supplementation on the human monocyte gene expression profile [18]. In our study, cDNA microarrays were used to identify gene expression changes in the white blood cells of healthy human volunteers after chronic consumption of either orange juice or purified hesperidin. This comparison aims to determine to what extent hesperidin is implicated in the nutrigenomic effects of orange juice.

## Materials and Methods

The protocol for this trial and supporting CONSORT checklist are available as supporting information; see Checklist S1 and Protocol S1.

### The human intervention study

#### Subjects

Twenty-four overweight male volunteers, 50–65 years of age, were recruited by newspaper advertisements. All subjects were healthy and had no evidence of chronic disease. Exclusion criteria were as follows: use of medication, use of antioxidants or vitamin supplements, smoking, alcohol consumption (>20 g alcohol/d), intense physical activity (>5 h/wk), intestinal disorders and vegetarianism. Subjects presenting with a high consumption of flavonoid-rich beverages, such as tea, herb tea, coffee, wine, cocoa and fruit juice, were also excluded when the daily consumption of one or more of these products exceeded 500 ml (as estimated from food frequency questionnaire). The study was approved by and performed under the guidelines of the French Human Ethics Committee of the South East VI. Written informed consent was obtained from each of the subjects before commencement of the study. All subjects completed the study except one who was excluded because of concurrent disease. Each volunteer enrolled in the study received 1130 euros. Clinical trial, settings and data collection were performed at the clinical research unit.

#### Test drinks and products

The details regarding these products were largely described previously [1]. Briefly, orange juice from concentrate was provided by the Florida Department of Citrus (Lake Alfred, FL, USA) and its hesperidin content was 292 mg/500 ml, corresponding to the daily administered dose. The control drink had a sugar composition similar to that of orange juice (**Table S1**), which made the two beverages isocaloric. Hesperidin capsules were filled with the orange bioflavonoid complex (OBC 90%) containing 99.2% hesperidin (Nutrafur, Murcia, Spain). Each capsule contained 146 mg of OBC, which corresponds to the amount of hesperidin in 250 ml orange juice. Placebo capsules consisted of starch (146 mg/capsule) and were visually identical to the hesperidin capsules.

#### Study design

**Figure 1:**
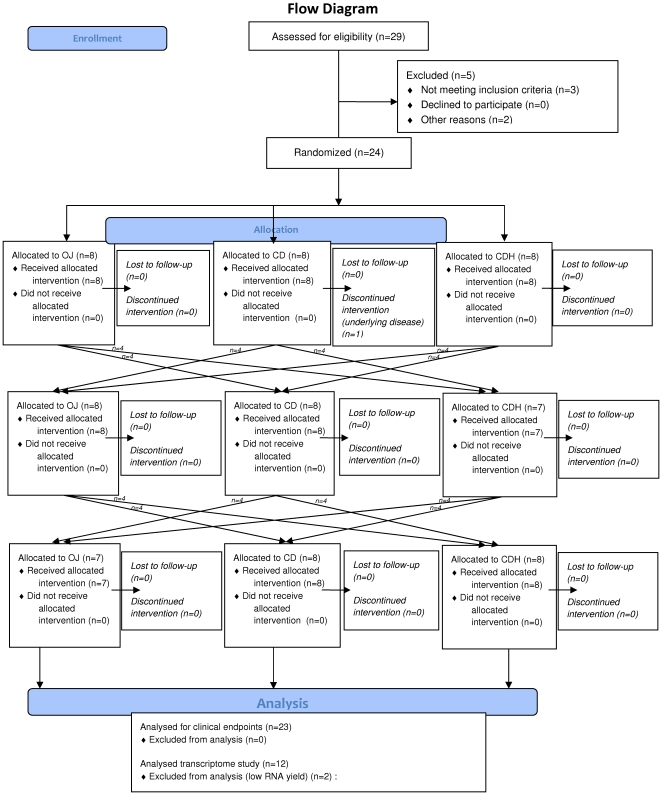
The flow diagram of the study.

The primary aim of the present randomized, cross-over study was to assess the effect of orange juice and hesperidin on endothelial and vessel function for which the results have been published elsewhere [1], and the second outcome was to examine the contribution of hesperidin in changes in gene expression in circulating blood cells induced by orange juice consumption. Briefly, the clinical study was a controlled, randomized, crossover, 4-week dietary intervention trial with three treatment groups: control drink+placebo, control drink+hesperidin and orange juice. For the orange juice intervention, the trial was open, but it was double-blinded for control drink+placebo and control drink+hesperidin. A 3-week washout period was applied between each treatment. A flow diagram of the study design is presented in **Figure 1**. The order of administration was determined using computer-generated random numbers. The randomization is based on a randomized Latin square to form six possible combinations from the three treatments, and the six groups were balanced (4 subjects/group). Randomization and implementation procedures were performed by a person from the clinical investigation unit not involved in the clinical trial.

Recruitment started on June 2007 and ended on September 2007, and the follow-up ended on February 2008. Each subject was successively assigned to three periods of four-week dietary treatments with a daily consumption of either: 1) 500 ml of a control drink plus 2 placebo capsules, 2) 500 ml of the control drink plus 2 capsules of pure hesperidin or 3) 500 ml of orange juice, which naturally provides 292 mg hesperidin. At home, subjects were instructed to divide the total daily dose into two equal intakes, one with breakfast and the other at lunchtime. On the first and last day of each experimental period, cardiovascular measurements (blood pressure and microvascular reactivity) and blood sample collections were performed on fasted volunteers. The calculation of the number of subjects to include in the trial was based on a statistical power ≥0.8, considering variability in laser Doppler recordings observed in previous experiments conducted in our laboratory to be between 16 and 21% and to have mean significant difference of at least 15%. Statistical analyses have performed using The PROC MIXED (SAS Institute) procedure by using a mixed linear model as detailed in [1]. Among the 24 volunteers enrolled in the randomized controlled cross-over study, 12 volunteers, selected by drawing lots at the beginning of the trial, participated in the present nutrigenomic study. This number of volunteers has been chosen regarding several previous nutrigenomic studies in clinical trials in which the number of volunteers varied from 3 to 15 [15], [16], [18], [19].

### Blood samples

Blood samples were collected, after an overnight fasting period, from the 10 selected volunteers at the end of each 4-week dietary intervention (control drink+placebo; control drink+hesperidin; orange juice). White blood cells were obtained using the LeukoLOCK total RNA isolation system (Ambion, Austin, USA) as per the manufacturer's instructions. In brief, 9 ml of blood samples with EDTA were passed through a LeukoLOCK Filter that captures leukocytes (white blood cells). The filters were flushed with phosphate-buffered saline (PBS) followed by RNAlater solution to stabilize the leukocyte RNA and stored at −80°C until used.

### RNA isolation

RNA was isolated using the LeukoLOCK total RNA isolation system (Ambion, Austin, USA) as recommended by the manufacturer. Captured cells were disrupted from the LeukoLOCK filter in a guanidine thiocyanate-based solution that rapidly releases total RNA. A brief proteinase K treatment was performed to degrade cellular proteins. Total RNA was purified using magnetic beads, and a DNase treatment was performed to remove genomic DNA. The quality of obtained total RNA was verified by electrophoresis in a 1% agarose gel, and the RNA concentration was determined (Thermo Scientific NanoDropTM). Using this approach, we obtained good quality total RNA from 30 samples at an average concentration of 75 ng/µl.

### RNA labeling

Five micrograms of total RNA were used to synthesize fluorescently labeled cDNA using the Pronto ChipShot™ Direct Labeling Kit (Corning, Avon, France) according to the manufacturer's protocol. Briefly, a reverse transcription reaction was performed using 1 µL of random primer and 1 µL of oligo(dT) and then labeled with dyes (Cy™ Dye Post-labeling Reactive Dye Pack, GE Healthcare, Buckinghamshire, UK). The labeled cDNA was purified by application to an equilibrated filter cartridge using the ChipShot™ Membrane Clean-Up System (Promega, Madison, WI, USA) following the manufacturer's recommendations. Quantity and labeling efficiency of labeled cDNA were determined by quantifying the absorbance at 260, 550 and 650 nm using a spectrophotometer NanoDrop™ (Thermo Scientific, Palaiseau, France). Each RNA sample was labeled with the dyes swapped to eliminate artifacts resulting from variation in the different dyes' incorporation in the experimental and control samples.

### Microarrays hybridization

Human oligonucleotide microarrays were obtained from Operon (Operon, Cologne, Germany). The Operon Human Genome Array-Ready Oligo Set™ (AROS) Version 4.0 contains 35,035 oligonucleotide probes representing approximately 25,100 unique genes and 39,600 transcripts excluding control oligonucleotides. The human version 4.0 is constructed based on the Ensembl human database with full coverage of the NCBI human Refseq dataset. Hybridization of microarrays with fluorescently labeled cDNA was conducted in a Ventana hybridization system (Ventana Medical Systems, S.A, Illkirch, France) at 42°C for 8 h. Slides were subsequently washed twice in 2× SSC and 0.1× SSC at room temperature. Buffer remaining on the slide was removed by rapid centrifugation (4000 g, 15 sec). The fluorescence intensity was scanned using an Agilent Microarray Scanner G2505B (Agilent Technologies, Inc., Santa Clara, CA, USA). Microarray data are being deposit in conformity to MIAME guidelines in the public ArrayExpress Archive database of microarray gene expression data at the European Bioinformatics Institute (http://www.ebi.ac.uk/arrayexpress/).

### Data analysis

Image analysis and statistical tests were performed as described [7]. The signal and background intensity values for each spot in both channels were obtained using ImaGene 6.0 software (Biodiscovery, Inc, Proteigene, Saint Marcel, France). The data were filtered using the ImaGene “empty spot” option that automatically flags and removes low-expressed and missing spots from the analysis. After log base 2 transformation, the data were corrected for systemic dye bias by Lowess normalization using GeneSight 4.1 software (BioDiscovery, El Segundo, USA). Statistical analyses were performed using the free R 2.1 software (http://www.r-project.org) after controlling for the variance of each gene. The data were analyzed using a standard Student's t-test to detect genes that were differentially expressed in the orange and hesperidin groups as compared to the control group. Probability values were adjusted using the Bonferroni correction for multiple testing at 1% to eliminate false positives. Genes selected by these criteria are referred to as “differentially expressed genes”.

Gene ontology (GO) annotations of biological processes for differentially expressed genes were conducted using GOstat (http://gostat.wehi.edu.au) [20]. This program determines all annotated GO terms for all genes and applies a Fisher's Exact Test to evaluate whether the observed difference is significant or not. The Benjamini and Hochberg correction was also performed to control the false discovery rate.

## Results

### Volunteers' baseline characteristics

Subjects enrolled in the clinical trial study were healthy men (56±1 years old) who were slightly overweight (average BMI of 27.4±0.3 kg/m^2^, **Table 1**). Subjects ranged from normal to mildly hyperlipidemic, as calculated from the baseline values for plasma cholesterol and triglyceride concentrations, and two-thirds of the subjects were normotensive. The dietary survey, performed before the study and at the end of each experimental period, did not reveal any significant changes in food habits throughout the intervention study. No significant changes in anthropometric and biochemical parameters (blood lipids, glycemia and insulinemia) were observed during the entire study period as we previously reported [1].

**Table 1:** Baseline characteristics of the entire study population on the screening day^1^.

	Mean ± SEM	Range
Age (y)	56±1	51–63
Body Mass index (kg/m^2^)	27.4±0.3	25.2–30.5
Systolic Blood Pressure (mmHg)	134±3	108–169
Diastolic Blood Pressure (mmHg)	82±2	64–109
Triglycerides (mmol/l)	1.42±0.14	0.50–2.60
Total Cholesterol (mmol/l)	5.65±0.17	4.00–6.70
HDL-cholesterol (mmol/l)	1.49±0.05	1.02–1.97
Fasting Glucose (mmol/l)	5.48±0.17	4.60–8.80

1 n = 24. CRP, C-reactive protein.

### Gene expression profiles in leukocytes

**Figure 2:**
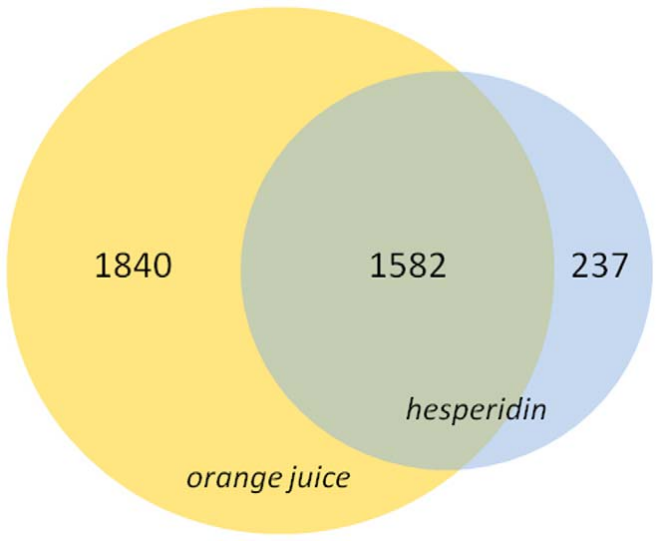
A Venn diagram of the number of differentially expressed genes in human blood leukocytes after a 4-week consumption of orange juice, hesperidin or both. Expression of these genes was significantly different from the control drink consumption group.

RNA from the white blood cells of ten fasted volunteers was used for the gene microarray study. After paired statistical analysis followed by false positive correction of the gene expression profiles, modification of the gene expression profile was observed in blood cells from volunteers that consumed orange juice or hesperidin+control drink in comparison to the placebo+control drink group. These differentially expressed genes were divided into three groups: 1) genes that were differentially expressed in the orange juice consumption group, 2) genes with differential expression in response to hesperidin consumption and 3) genes that were differentially expressed in response to both the orange juice and hesperidin treatments. Using this approach, we identified 3,422 genes as differentially expressed by orange juice (**Table S2**) and 1,819 genes by hesperidin (**Table S3**). Among the genes modulated by orange juice consumption, 1,706 were up-regulated and 1,716 genes were down-regulated, with an average fold change of 1.42 and −1.30, respectively. For the hesperidin group, 1,033 genes were upregulated, with an average fold change of 1.34, and 786 genes were down-regulated, with an average fold change of −1.25. Interestingly, 1,582 of these genes overlapped with the same direction of change between the 2 groups, being similarly down- or up-regulated in the response to both orange juice and hesperidin (**Figure 2**). Nevertheless, for hesperidin group, a slightly lower magnitude of gene expression difference was observed, as revealed by a reduction of approximately 20–30% of the fold change when compared to the orange juice group.

To determine the roles of the differentially expressed genes, genes were classified into functional categories according to their biological process gene ontology annotations using GOstat software. Over-represented gene ontologies (GO) for the two groups are shown in **Table S4** and **Table S5**. Among the GO terms identified for the orange juice group, more than 70% were also identified for the hesperidin group. Differentially-expressed genes, modulated by both orange juice and hesperidin consumption, are involved in different biological processes with the most over-represented processes being signal transduction, cell adhesion, immune response, cell proliferation, chemotaxis and lipid metabolism. Some of these processes were over-represented in the up- or down-regulated genes, (e.g., chemotaxis, cytoskeleton organization or biogenesis), and the majority of genes were downregulated. On the other hand, genes implicated in processes such as the inflammatory response or the I-kappaB kinase/NF-kappaB cascade were overrepresented in the upregulated genes.

## Discussion

In this study, we demonstrated that the consumption of orange juice or hesperidin for 4-weeks in healthy men affected blood leukocyte gene expression profiles. Several of these genes are modulated by both orange juice and hesperidin. Thus, it is conceivable that hesperidin, a major orange phenol, contributes to the biological effects of its food carrier. Several of observed changes in gene expression could be described as anti-inflammatory and anti-atherogenic responses to these dietary interventions.

**Figure 3:**
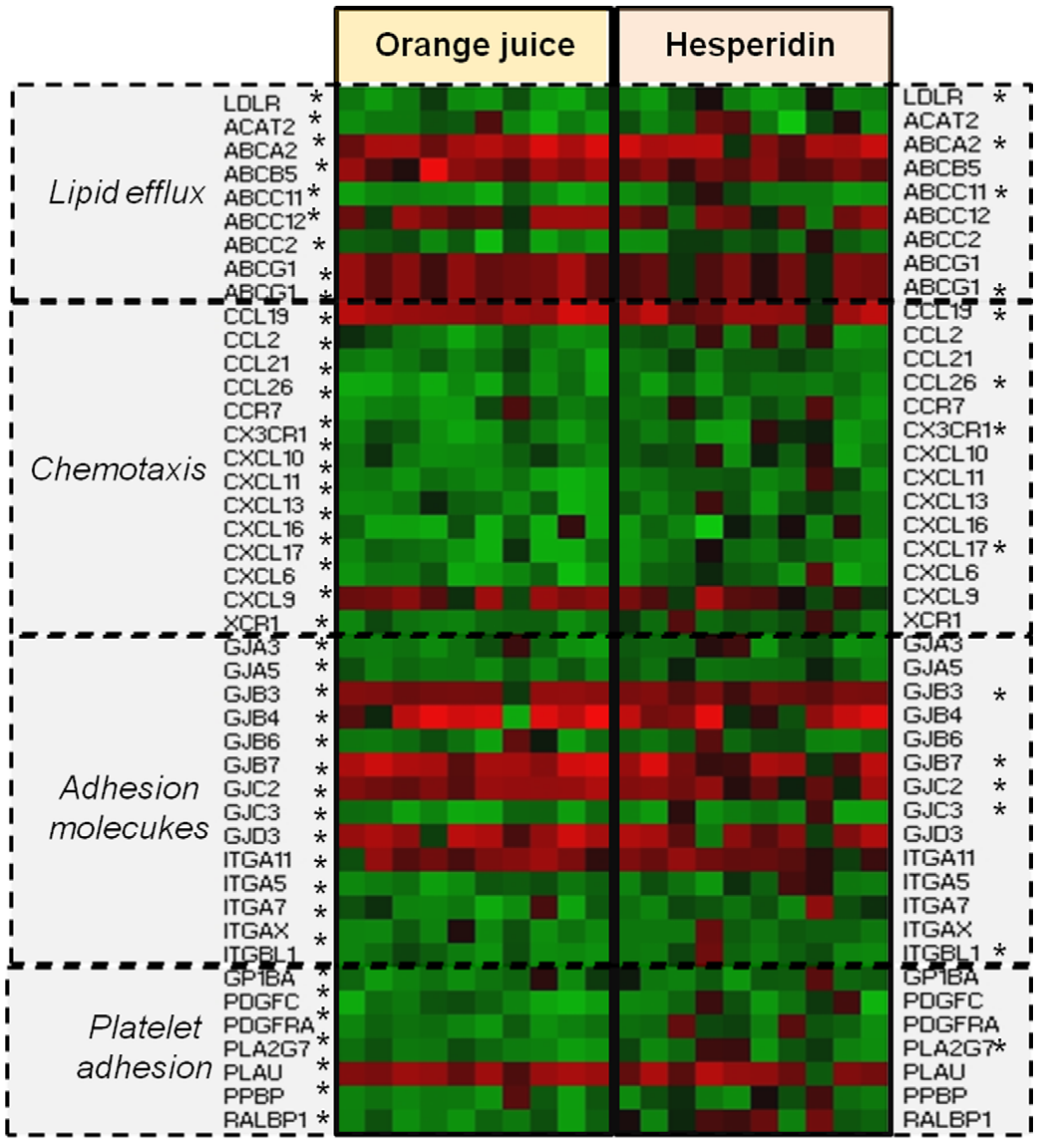
A heat-map of atherosclerosis-related gene expression changes after the consumption of orange juice or hesperidin in all 10 subjects. Red indicates up-regulation and green indicates down-regulation. Statistically significant genes, after a p-value adjustment to minimize false positives using the Bonferroni correction for multiple testing, are represented by an asterisk.

**Figure 4:**
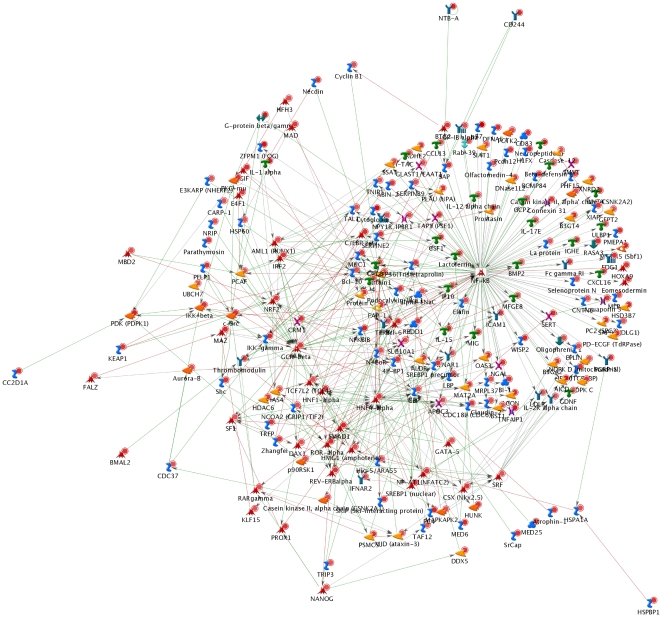
A representative biological network based on the differentially expressed genes of the orange juice group using MetaCore™ network software and the Analyze Network algorithm. The network shown is the NF-κB network. Dots in the right corner of a gene indicate differential expression.

Gene Ontology analysis of differentially expressed genes revealed that both orange juice and hesperidin modulated the expression of numerous genes that are potentially implicated in the processes of inflammation and atherosclerosis development. Among them are genes encoding chemokines, such as CCL26, CX3CR1, or CXCL17, for which expression was downregulated (**Figure 3**). Chemokines are chemoattractant cytokines that regulate leukocyte trafficking and activation [21]. CX3CR1 is expressed in monocytes and is involved in their recruitment to atherosclerotic arteries. Increased expression of CX3CR1 has been observed in foam cells in human atherosclerotic arteries but not in normal human arterial tissue [22]. Furthermore, a deletion of CX3CR1 in apoE−/− mice decreased macrophage accumulation [23]. Both orange juice and hesperidin also downregulated the expression of CCL26, gene expressed in human monocytic cells that is regulated by IL-4 [24]. Interestingly, our transcriptome analysis also showed decreased expression of IL4 in response to both orange juice and hesperidin. Orange juice modulated the expression of 9 other genes encoding chemokines and their receptors. In particular, it downregulated the expression of monocyte chemoattractant protein-1 (MCP-1 or CCL2), a strong chemoattractant involved in monocyte/macrophage migration and infiltration. Plasma levels of CCL2 are higher in patients with peripheral arterial disease [25]. Inactivation of the MCP-1 gene in atherosclerosis mouse models resulted in a significant decrease in lesion size together with lower macrophage infiltration [26]. The observed downregulation of CCL2 could be in agreement with a report from a previous clinical trial that showed a decrease in CCL2 levels after chronic consumption of a polyphenol-rich sparkling wine [27]. Orange juice consumption also decreased the expression of other genes known to be highly expressed in human atheromas throughout all stages of plaque development, such as CCL21, CXCL11 and CXCL16 [28], [29]. These genes encoding chemokines are regulated by the NFkB transcription factor. Our microarray analysis also revealed that both orange juice and hesperidin upregulated the expression of the inhibitor of NFkB (NFkBIB). This observation suggests that upregulation of the expression of NFkBIB could, in turn, inhibit NFkB activity and be related to the observed downregulation of the expression of genes encoding the chemokines. Interestingly, a bioinformatics analysis revealed that NFkB (**Figure 4**) appeared as one of the most statistically relevant transcription factors for which activity could be modulated by both orange juice and hesperidin. This finding seems to be in agreement with previous studies suggesting that flavonoids might modulate the activity of this transcription factor and the expression of NFkB-dependent genes [30]. In agreement with the possible impact of orange juice and hesperidin on NFkB-dependent genes, we also observed changes in the expression of several genes encoding adhesion molecules (**Figure 3**). The interaction of circulating blood cells with the endothelium is the first event in atheroma plaque formation and requires the participation of different adhesion proteins. Both orange juice and hesperidin downregulated expression of CD80. This regulator of T-cell activation was found to be upregulated during the monocyte-endothelial cell interaction [31] as in patients with CVD [32]. Interestingly, both orange juice and hesperidin induced expression of BCL6, a transcriptional repressor that prevents the expression of CD80 by binding to its promoter region [33]. Furthermore, orange juice and hesperidin also induced the expression of another cell adhesion molecule, CEACAM3 which inactivation induced neutrophil adhesion to endothelial cells [34]. The transcriptome study also revealed the downregulation of ITGBL1 by both orange juice and hesperidin, while only orange juice decreased the expression of several other genes encoding integrins, such as ITGA5, ITGA7 and ITGAX. It has been shown that ITGAX deficiency reduced the firm arrest of monocytes on vascular cells, monocyte/macrophage accumulation in intimae and consequently, reduced atherosclerosis development in apoE−/− mice fed a high-fat diet [35]. Besides the observed modification in expression of the integrin genes, we also observed expression modulation of genes encoding connexins, known as gap junction adhesion molecules. For example, both orange juice and hesperidin downregulated the expression of connexin 31.3 while connexins 30, 40 and 46 were only downregulated by orange juice. In response to orange juice or hesperidin consumption, the combined expression regulation of genes encoding chemokines and their receptors and adhesion molecules suggests a lower recruitment and infiltration of circulating cells to the vascular wall.

From our study, a large subset of genes implicated in the processes of lipid metabolism and transport has been identified as differentially expressed in response to orange juice and hesperidin consumption (**Figure 3**). In particular, we observed a significant downregulation of LDLR, the receptor responsible for LDL binding and internalization in macrophages. Inside the cell, free cholesterol is converted to a cholesteryl ester and forms lipid droplets due to acylCoA:cholesterol acyltransferase (ACAT) activity. In our study, ACAT2 was downregulated by orange juice consumption, suggesting a potential decrease in lipid droplet accumulation, which could reduce foam cell formation. This result could be in agreement with a previous study which showed that flavonoid from grapefruit, naringenin, inhibits ACAT2 activity [36]. Furthermore, macrophage intracellular cholesterol is regulated by membrane transporters of the ATP-binding cassette (ABC) superfamily. These proteins are implicated in reverse cholesterol transport from macrophages, which represents a potential protective effect against foam cell formation, the hallmark of atherosclerosis [37]. ABCA2 is one of the most upregulated genes by both orange juice and hesperidin. Moreover, both orange juice and hesperidin also induced expression of the ABCF1 gene, while orange juice up-regulated other ABC-transporter genes, such as ABCG1 and ABCB5. Disrupting ABCG1 in mice was observed to promote the accumulation of excess cholesterol in macrophages [38]. Overall, the observed decrease in the expression of genes encoding the LDL receptor and ACAT2 together with an increase in the expression of genes regulating reverse cholesterol transport suggests that white blood cells would be less prone to differentiate into foam cells after 4 weeks of orange juice or hesperidin consumption.

A comparison of the global nutrigenomic profiles of orange juice and hesperidin revealed that hesperidin modulated a smaller number of genes and induced lower fold-changes. This difference in genomic effect is not surprising as we compared a complex beverage versus pure hesperidin administered with a control sugar drink. Nevertheless, the 53% of genes modulated by orange juice also constitute the potential molecular targets of hesperidin, which suggests that hesperidin plays an important role in the genomic effects of this beverage. This finding represents the first evidence of a role for a polyphenolic compound in the gene-mediated effects of a complex food in human. Changes in leukocyte gene expression mediated by hesperidin were observed in fasted subjects while hesperidin metabolites were no longer present in blood circulation [39]. This persistent effect at the genomic level could be regarded as an adaptation of leukocytes to continuous high-flavanone exposure.

**Figure 5:**
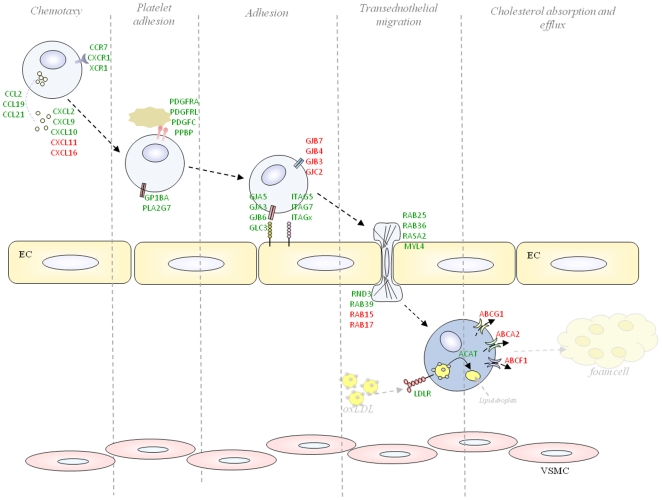
A summary of the impact of orange juice and/or hesperidin on the expression of genes implicated in the initial processes of atherosclerosis development. Changes in gene expression are indicated by red for up-regulation and green for down-regulation. EC, endothelial cell; VSMC, muscular smooth muscle cell.

In summary, this study reveals that numerous genes modulated by orange juice and hesperidin in blood cells are involved in processes that regulate interactions with the endothelium (adhesion, infiltration) and lipid accumulation (**Figure 5**). This nutrigenomic study shows that regular consumption of orange juice alters gene expression to a potentially protective cardiovascular profile. Furthermore, this work gives insight into the molecular mechanisms by which flavanones contribute to the vascular-protective effects of citrus fruits in humans.

## Supporting Information

Checklist S1 CONSORT checklist.
(DOC)

Protocol S1 Trial protocol.
(PDF)

Table S1 Main constituents and phytochemicals contents in the test drinks (500 ml) used in the study.
(DOC)

Table S2 List of differentially expressed genes after orange juice consumption.
(XLS)

Table S3 List of differentially expressed genes after hesperidin consumption.
(XLS)

Table S4 Gene onthology of differentially expressed genes after orange juice consumption (from Gostat; http://gostat.wehi.edu.au/).
(XLS)

Table S5 Gene onthology of differentially expressed genes after hesperidin consumption (from Gostat; http://gostat.wehi.edu.au/).
(XLS)

## References

[1] Morand C, Dubray C, Milenkovic D, Lioger D, Martin JF (2011). Hesperidin contributes to the vascular protective effects of orange juice: a randomized crossover study in healthy volunteers.. Am J Clin Nutr.

[2] Mink PJ, Scrafford CG, Barraj LM, Harnack L, Hong CP (2007). Flavonoid intake and cardiovascular disease mortality: a prospective study in postmenopausal women.. Am J Clin Nutr.

[3] Guarnieri S, Riso P, Porrini M (2007). Orange juice vs vitamin C: effect on hydrogen peroxide-induced DNA damage in mononuclear blood cells.. Br J Nutr.

[4] Ghanim H, Sia CL, Upadhyay M, Korzeniewski K, Viswanathan P (2010). Orange juice neutralizes the proinflammatory effect of a high-fat, high-carbohydrate meal and prevents endotoxin increase and Toll-like receptor expression.. Am J Clin Nutr.

[5] Schroeter H, Heiss C, Balzer J, Kleinbongard P, Keen CL (2006). (-)-Epicatechin mediates beneficial effects of flavanol-rich cocoa on vascular function in humans.. Proc Natl Acad Sci U S A.

[6] Widlansky ME, Duffy SJ, Hamburg NM, Gokce N, Warden BA (2005). Effects of black tea consumption on plasma catechins and markers of oxidative stress and inflammation in patients with coronary artery disease.. Free Radic Biol Med.

[7] Auclair S, Milenkovic D, Besson C, Chauvet S, Gueux E (2009). Catechin reduces atherosclerotic lesion development in apo E-deficient mice: a transcriptomic study.. Atherosclerosis.

[8] Ludwig A, Lorenz M, Grimbo N, Steinle F, Meiners S (2004). The tea flavonoid epigallocatechin-3-gallate reduces cytokine-induced VCAM-1 expression and monocyte adhesion to endothelial cells.. Biochem Biophys Res Commun.

[9] Vafeiadou K, Vauzour D, Lee HY, Rodriguez-Mateos A, Williams RJ (2009). The citrus flavanone naringenin inhibits inflammatory signalling in glial cells and protects against neuroinflammatory injury.. Arch Biochem Biophys.

[10] Garcia-Conesa MT, Tribolo S, Guyot S, Tomas-Barberan FA, Kroon PA (2009). Oligomeric procyanidins inhibit cell migration and modulate the expression of migration and proliferation associated genes in human umbilical vascular endothelial cells.. Mol Nutr Food Res.

[11] de Boer VC, van Schothorst EM, Dihal AA, van der Woude H, Arts IC (2006). Chronic quercetin exposure affects fatty acid catabolism in rat lung.. Cell Mol Life Sci.

[12] Spencer JP (2010). Beyond antioxidants: the cellular and molecular interactions of flavonoids and how these underpin their actions on the brain.. Proc Nutr Soc.

[13] Camargo A, Ruano J, Fernandez JM, Parnell LD, Jimenez A (2010). Gene expression changes in mononuclear cells in patients with metabolic syndrome after acute intake of phenol-rich virgin olive oil.. BMC Genomics.

[14] Chanet A, Milenkovic D, Deval C, Potier M, Constans J (2011). Naringin, the major grapefruit flavonoid, specifically affects atherosclerosis development in diet-induced hypercholesterolemia in mice.. J Nutr Biochem.

[15] van Erk MJ, Blom WA, van Ommen B, Hendriks HF (2006). High-protein and high-carbohydrate breakfasts differentially change the transcriptome of human blood cells.. Am J Clin Nutr.

[16] Bouwens M, Grootte Bromhaar M, Jansen J, Muller M, Afman LA (2010). Postprandial dietary lipid-specific effects on human peripheral blood mononuclear cell gene expression profiles.. Am J Clin Nutr.

[17] Ardigo D, Gaillard CA, Braam B (2007). Application of leukocyte transcriptomes to assess systemic consequences of risk factors for cardiovascular disease.. Clin Chem Lab Med.

[18] Boomgaarden I, Egert S, Rimbach G, Wolffram S, Muller MJ (2010). Quercetin supplementation and its effect on human monocyte gene expression profiles in vivo.. Br J Nutr.

[19] Bouwens M, Afman LA, Muller M (2007). Fasting induces changes in peripheral blood mononuclear cell gene expression profiles related to increases in fatty acid beta-oxidation: functional role of peroxisome proliferator activated receptor alpha in human peripheral blood mononuclear cells.. Am J Clin Nutr.

[20] Beissbarth T, Speed TP (2004). GOstat: find statistically overrepresented Gene Ontologies within a group of genes.. Bioinformatics.

[21] Weber C, Schober A, Zernecke A (2004). Chemokines: key regulators of mononuclear cell recruitment in atherosclerotic vascular disease.. Arterioscler Thromb Vasc Biol.

[22] Wong BW, Wong D, McManus BM (2002). Characterization of fractalkine (CX3CL1) and CX3CR1 in human coronary arteries with native atherosclerosis, diabetes mellitus, and transplant vascular disease.. Cardiovasc Pathol.

[23] Combadiere C, Potteaux S, Rodero M, Simon T, Pezard A (2008). Combined inhibition of CCL2, CX3CR1, and CCR5 abrogates Ly6C(hi) and Ly6C(lo) monocytosis and almost abolishes atherosclerosis in hypercholesterolemic mice.. Circulation.

[24] Stubbs VE, Power C, Patel KD (2010). Regulation of eotaxin-3/CCL26 expression in human monocytic cells.. Immunology.

[25] Hoogeveen RC, Morrison A, Boerwinkle E, Miles JS, Rhodes CE (2005). Plasma MCP-1 level and risk for peripheral arterial disease and incident coronary heart disease: Atherosclerosis Risk in Communities study.. Atherosclerosis.

[26] Ni W, Egashira K, Kitamoto S, Kataoka C, Koyanagi M (2001). New anti-monocyte chemoattractant protein-1 gene therapy attenuates atherosclerosis in apolipoprotein E-knockout mice.. Circulation.

[27] Vazquez-Agell M, Sacanella E, Tobias E, Monagas M, Antunez E (2007). Inflammatory markers of atherosclerosis are decreased after moderate consumption of cava (sparkling wine) in men with low cardiovascular risk.. J Nutr.

[28] Damas JK, Smith C, Oie E, Fevang B, Halvorsen B (2007). Enhanced expression of the homeostatic chemokines CCL19 and CCL21 in clinical and experimental atherosclerosis: possible pathogenic role in plaque destabilization.. Arterioscler Thromb Vasc Biol.

[29] Apostolakis S, Papadakis EG, Krambovitis E, Spandidos DA (2006). Chemokines in vascular pathology (review).. Int J Mol Med.

[30] Hishikawa K, Nakaki T, Fujita T (2005). Oral flavonoid supplementation attenuates atherosclerosis development in apolipoprotein E-deficient mice.. Arterioscler Thromb Vasc Biol.

[31] Wang P, Liu Z, Wu C, Zhu B, Wang Y (2008). Evaluation of CD86/CD28 and CD40/CD154 pathways in regulating monocyte-derived CD80 expression during their interaction with allogeneic endothelium.. Transplant Proc.

[32] Dopheide JF, Sester U, Schlitt A, Horstick G, Rupprecht HJ (2007). Monocyte-derived dendritic cells of patients with coronary artery disease show an increased expression of costimulatory molecules CD40, CD80 and CD86 in vitro.. Coron Artery Dis.

[33] Niu H, Cattoretti G, Dalla-Favera R (2003). BCL6 controls the expression of the B7-1/CD80 costimulatory receptor in germinal center B cells.. J Exp Med.

[34] Skubitz KM, Skubitz AP (2008). Interdependency of CEACAM-1, -3, -6, and -8 induced human neutrophil adhesion to endothelial cells.. J Transl Med.

[35] Wu H, Gower RM, Wang H, Perrard XY, Ma R (2009). Functional role of CD11c+ monocytes in atherogenesis associated with hypercholesterolemia.. Circulation.

[36] Nahmias Y, Goldwasser J, Casali M, van Poll D, Wakita T (2008). Apolipoprotein B-dependent hepatitis C virus secretion is inhibited by the grapefruit flavonoid naringenin.. Hepatology.

[37] Pennings M, Meurs I, Ye D, Out R, Hoekstra M (2006). Regulation of cholesterol homeostasis in macrophages and consequences for atherosclerotic lesion development.. FEBS Lett.

[38] Oram JF, Vaughan AM (2006). ATP-Binding cassette cholesterol transporters and cardiovascular disease.. Circ Res.

[39] Manach C, Williamson G, Morand C, Scalbert A, Remesy C (2005). Bioavailability and bioefficacy of polyphenols in humans. I. Review of 97 bioavailability studies.. Am J Clin Nutr.

