# Supplementary material for: Hesperidin Displays Relevant Role in the Nutrigenomic Effect of Orange Juice on Blood Leukocytes in Human Volunteers: A Randomized Controlled Cross-Over Study
Source: PLoS One. 2011 Nov 16;6(11):e26669. doi: 10.1371/journal.pone.0026669 (PMC3217928; doi:10.1371/journal.pone.0026669)
Supplement: Protocol S1 — Trial protocol. (PDF) [file pone.0026669.s002.pdf]

INRA

CHU CLERMONT FERRAND

CIC Inserm CLERMONT-Fd

Effets de la consommation de jus d'orange sur la protection vasculaire et la fonction immunitaire : étude clinique sur la contribution spécifique des flavanones des agrumes

Etude FLORIDE

Code promoteur : RBHP 2006 Dubray
N° IDRCB : 2006-A00653-48
Version n° 1 du 20 décembre 2006

Etude proposée par l'INRA :
Christine MORAND
Unité de Nutrition Humaine (UNH) - Centre de Recherche de Clermont-Ferrand -Theix
63122 Saint-Genès-Champanelle
Tél. +33 (0)4 73 62 47 87 (direct)   +33 (0)4 73 62 42 38 (secrétariat)
Fax 33 (0)4 73 62 47 55 E-mail: cmorand@clermont.inra.fr

Promoteur :
Centre Hospitalier Universitaire de Clermont-Ferrand
Place Henri Dunant - BP 69
63003 Clermont-Ferrand Cedex 1
Tél : 04.73.750.750

Investigateur principal :
Pr Claude DUBRAY, Centre de Pharmacologie Clinique, Inserm CIC-501

Investigateurs :
DrChristian DUALE, Centre de Pharmacologie Clinique, Inserm CIC-501
Dr Gisèle PICKERING, Centre de Pharmacologie Clinique, Inserm CIC-501

Partenaires scientifiques :
Pr Marie-Paule VASSON, responsable EA 2416, Faculté de Pharmacie, Université d'Auvergne,
Christine MORAND, UNH
Christian DEMIGNE, UNH
André MAZUR, UNH
Dragan MILENKOVIC, UNH
Augustin SCALBERT, UNH
Anne DE LA TORRE, Université d'Auvergne, Faculté de Pharmacie

Lieu de la recherche :
Centre d'Investigation Clinique (CIC)
Bâtiment 3C
58 rue Montalembert
63003 Clermont-Ferrand cedex 1
Tél : 04.73.17.84.10

SOMMAIRE

RESUME **4**

LISTE DES ABREVIATIONS **5**

1 JUSTIFICATION SCIENTIFIQUE DE L'ETUDE **7**

2 OBJECTIFS DE L'ETUDE **12**

2.1 OBJECTIF PRINCIPAL 12
2.2 OBJECTIFS SECONDAIRES 12

3 CRITERES D'EVALUATION **12**

3.1 CRITERE D'EVALUATION PRINCIPAL 13
3.2 CRITERES D'EVALUATION SECONDAIRES 18
3.2.1 Détermination des biomarqueurs associés au risque cardiovasculaire 18
3.2.2 Evaluation du phénotype des cellules immunitaires et leurs fonctions 19
3.2.3 Evaluation de l'équilibre acido-basique 21
3.2.4 Analyse transcriptomique des cellules PBMC isolées 21

4 METHODOLOGIE DE LA RECHERCHE **23**

4.1 DEROULEMENT DE L'ETUDE 23
4.2 RECRUTEMENT DES VOLONTAIRES 28
4.3 PROCEDURE DE RANDOMISATION DES SUJETS 29
4.4 DUREE DE PARTICIPATION DES SUJETS DE L'ETUDE 30
4.5 PROCEDURE DE LA VERIFICATION DE LA COMPLIANCE DES SUJETS 30
4.6 DONNEES A RECUEILLIR LORS DE DIFFERENTES VISITES 30
4.6.1 Données à recueillir lors de la visite de sélection : 30
4.6.2 Données à recueillir lors de la visite d'inclusion (V1) 31
4.6.3 Données à recueillir lors des visites 2, 4 et 6 (début de période) 32
4.6.4 Données à recueillir lors des visites 3, 5 et 7 (fin de période) 33

5 DESCRIPTION DE LA POPULATION DE L'ETUDE **34**

5.1 CRITERES D'INCLUSION 34
5.2 CRITERES DE NON-INCLUSION 34
5.3 CRITERES D'ORDRE NUTRITIONNELS SUPPLEMENTAIRES POUR L'INCLUSION 35

6 RESUME DES BENEFICES ET DES RISQUES PREVISIBLES POUR LES VOLONTAIRES **35**

7 PROCEDURES DE DECLARATION DES EVENEMENTS INDESIRABLES GRAVES **36**

8 STATISTIQUES **36**

8.1 CALCUL DU NOMBRE DE SUJETS A INCLURE 36
8.2 DESCRIPTION DES METHODES STATISTIQUES UTILISEES POUR L'ANALYSE DES DONNEES 37

9 CONSIDERATIONS PRATIQUES **38**

9.1 DECLARATIONS OFFICIELLES 38
9.2 CONTROLE ET ASSURANCE DE LA QUALITE 38
9.3 ARCHIVAGE DES DOCUMENTS DE L'ETUDE 38
9.4 CONTRAT D'ASSURANCE 38
9.5 CALENDRIER PREVISIONNEL DE L'ETUDE 39
9.6 REGLES RELATIVES A LA PUBLICATION 39

REFERENCES **40**

Paraphe du volontaire avec la mention « lu et compris »

LISTE DES ANNEXES **44**

Paraphe du volontaire avec la mention « lu et compris »

Résumé

Les études épidémiologiques ont fermement établi les effets sur la santé d'une consommation élevée de fruits et légumes et notamment en ce qui concerne la prévention des maladies cardiovasculaires. Les agrumes sont les fruits les plus largement consommés à travers le monde, essentiellement sous forme de jus. Les agrumes sont riches en vitamine C mais aussi en divers phytomicronutriments dont les plus abondants sont les polyphénols. Plusieurs études *in vitro* et sur modèles animaux suggèrent que les polyphénols des agrumes seraient impliqués dans les effets protecteurs des agrumes vis-à-vis des maladies cardiovasculaires. Toutefois, les bénéfices en matière de santé associés à la prise de ces polyphénols ne sont pas connus et jusqu'à présent aucune étude clinique n'a été réalisée pour déterminer la contribution des polyphénols des agrumes dans cette protection.

Les principaux objectifs de ce projet sont (1) de caractériser les effets en prise unique et en prises répétées d'une consommation de jus d'orange sur la protection vasculaire et sur la réponse immunitaire et (2) d'évaluer le rôle spécifique des polyphénols présents dans les agrumes dans cette protection. Pour cela il sera réalisé une étude clinique, randomisée et en cross over, chez des hommes sains de 50 à 65 ans présentant un léger surpoids. Ces volontaires, qui continueront à suivre leur alimentation habituelle devront consommer quotidiennement pendant 4 semaines (1) du jus d'orange ou (2) une boisson contrôle isocalorique ou bien (3) cette même boisson supplémentée avec une dose de polyphénols comparable à celle apportée par le jus d'orange.

La fonction vasculaire sera suivie en mesurant à la fois des paramètres systémiques et fonctionnels. En raison de relation avec les pathologies vasculaires, certains paramètres lipidiques seront mesurés dans le plasma ainsi que des biomarqueurs du stress oxydant, de l'inflammation et de l'activation endothéliale. La fonction endothéliale sera évaluée en mesurant la réactivité microvasculaire sous-cutanée par laser Doppler en réponse à l'application locale d'acétylcholine ou de nitroprussiate de sodium par iontophorèse. Ces effets seront mesurés à jeun et en période post prandiale. Les impacts d'une consommation prolongée de jus d'orange ou de polyphénols d'agrumes purifiés sur la fonction immunitaire seront recherchés en mesurant des marqueurs spécifiques. A partir des cellules mononuclées issues du sang des volontaires ayant consommés chacune des 3 boissons pendant 4 semaines, une étude transcriptomique (microarrays) sera aussi réalisée. Cette approche, en mettant en

Paraphe du volontaire avec la mention « lu et compris »

évidence des modifications de l'expression des gènes, devrait permettre d'apporter des données nouvelles sur les effets métaboliques et les cibles moléculaires des polyphénols. Enfin, les conséquences d'une consommation régulière de jus d'orange sur l'équilibre acido-basique, lui-même étroitement lié à la santé osseuse, seront recherchées.

Liste des abréviations

mA - µA : milliampère - microampère
Ach : Acétylcholine
ADN_c : Acide Désoxyribonucléique complémentaire
ALAT : Alanine Aminotransférase
ARN : Acide Ribonucléique
ASAT : Aspartate minotransférase
Ca : Calcium
CD-25 : Antigène CD-25
CHU : Centre Hospitalier Universitaire
CIC : Centre d'Ivestigation Clinique
CML : Cellules Musculaires Lisses
CPP : Comité de Protection des Personnes
CRP : C Réactive Protein
DGS : Direction Générale de la Santé
EDTA : Ethylène Diamine Tétra-Acétique
EGFP : Enhanced green fluorescent protein
ELISA : Enzyme linked ImmunoSorbent Assay
FMD : Flow Mediated Dilation
FRAP : Ferric Reducing Activity of Plasma
γGT : γ–Glutamyl Transférase
H2O2 : Peroxyde d'hydrogène
HDL : High Density Lipoprotein
HPLC : Chromatographie Liquide Haute Performance
IFN: Interféron
IL : Interleukine
INRA : Institut National de Recherche Agroalimentaire
K : Potassium
LC-MS : Chromatographie Liquide – Spectrométrie de masse
LD : Laser Doppler
LDH : lactate deshydrogénase
Mg : Magnésium
NADPH : Nicotinamide Adénine Dinucleotide Phosphate reduced
NK : Natural Killer
NO : Oxyde Nitrique
NPS : Nitroprussiate de sodium
PBMC : Peripheral Blood Mononuclear Cells
PCR : Réaction de Polymérisation en Chaîne
PMA : Phorbol Myristate Acétate
PMN : Neutrophiles Polynucléaires

Paraphe du volontaire avec la mention « lu et compris »

RNG : Réseau National des Génopoles
s-ICAM : soluble- IntraCellular Adhesion Molecule
s-VCAM : soluble- VascularCellular Adhesion Molecule
TG : Triglycérides
Th : Lymphocytes T helper
VHC : Virus de l'Hépatite C
VIH : Virus de l'Immunodéficience Humaine
vWF : Facteur de von Willebrand

Paraphe du volontaire avec la mention « lu et compris »

1 Justification scientifique de l’étude

Tous les types de maladies cardiovasculaires sont associés à des dysfonctionnements de l’endothélium qui ont des conséquences sur le tonus vasculaire mais qui jouent aussi un rôle crucial dans le déclenchement d’évènements inflammatoires conduisant au développement de l’athérosclérose (1). Il est établi que le recrutement de monocytes par l’endothélium vasculaire constitue une étape clé de l’athérogenèse et ce processus est facilité par l’attachement des monocytes aux cellules endothéliales sous l’effet de molécules d’adhésion produites par ces dernières. Au niveau des vaisseaux, ce phénomène s’accompagne de la transformation des monocytes en cellules spumeuses qui, en s’accumulant dans la paroi vasculaire, aboutit à la formation de la strie lipidique, contribuant ainsi au développement de la lésion d’athérosclérose. De plus, les cellules endothéliales produisent des facteurs vasoactifs qui influencent la fonction contractile des cellules musculaires lisses et par conséquent modulent le tonus vasculaire. Depuis que des études récentes ont montré que le dysfonctionnement endothélial constitue la première étape du processus d’athérosclérose, la mesure de la fonction endothéliale (par des paramètres fonctionnels et systémiques) apparaît comme un outil clé dans le dépistage de l’athérosclérose (2).

Les études épidémiologiques ont fermement établi le rôle protecteur de la consommation de fruits et de légumes, en particulier dans la prévention des pathologies cardiovasculaires (3-5). Pendant longtemps on a considéré que cet effet bénéfique était dû aux vitamines et caroténoïdes présents dans ces aliments et reconnus pour leurs propriétés antioxydantes. Cependant, les résultats décevants d’un certain nombre d’études d’intervention de grande envergure, qui ont montré aucune réduction de la mortalité générale et même parfois un plus grand risque cardiovasculaire (6), ont conduit les scientifiques à rechercher d'autres composés présents dans les fruits et les légumes potentiellement bénéfiques. C’est ainsi que ces dernières années une attention toute particulière a été accordée aux polyphénols, présents spécifiquement dans les produits végétaux et qui constituent les antioxydants les plus abondants dans notre alimentation.

Des études cliniques récemment publiées ont montré que plusieurs biomarqueurs durisque cardiovasculaire sont influencés par la consommation d'aliments riches en polyphénols, c'est en particulier le cas pour le thé, le vin, le cacao ou le soja (7). Les effets sur des biomarqueurs du stress oxydant, de la lipémie et de l'inflammation semblent jusqu'ici peu concluants. Par contre des effets plus constants ont été observés sur la fonction endothéliale et sur l'homéostase, et vont dans le sens d'une réduction du risque cardiovasculaire par des polyphénols, en accord avec les quelques études épidémiologiques déjà publiées sur des flavonoïdes et les lignanes (8). Toutes les études cliniques réalisées à ce jour, ont mis en œuvre des aliments ou des boissons contenant un mélange de différents polyphénols et dans ces conditions la nature exacte des composés les plus actifs demeure en grande partie inconnue. De plus, la plupart de ces études ont été réalisées à jeun alors que d'après les travaux sur la biodisponibilité des polyphénols, les concentrations maximales des polyphénols dans les tissus sont atteintes dans les quelques heures suivant leur ingestion (en phase postprandiale) (9). Il est donc important d'évaluer les effets des polyphénols non seulement à jeun mais également en phase postprandiale

Les perturbations du métabolisme se produisant en période postprandiale sont susceptibles de jouer un rôle important dans la progression des lésions d'athérosclérose, d'autant que l'état postprandial a été associé à un dysfonctionnement endothélial (10). Puisque les humains vivent en état postprandial quasiment tout au long de la journée, l'exposition prolongée à des facteurs alimentaires qui altèrent la fonction endothéliale ou retardent le rétablissement postprandial de l'endothélium pourrait négativement affecter le profil de risque cardiovasculaire (11). Des études récentes ont montré que chez des sujets sains, les concentrations plasmatiques en cytokines inflammatoires et en molécules d'adhésion augmentent de façon transitoire suite à l'ingestion d'un repas à forte teneur en graisses (12). Ceci montre que l'ingestion de graisses favorise un état pro-inflammatoire transitoire. C'est pourquoi nous proposons d'étudier si une ingestion quotidienne de jus d'orange ou des polyphénols purifiés issus d'oranges peut reverser l'effet délétère d'un repas à haute teneur en graisses sur l'activité endothéliale.

Les cellules immunitaires, telles que les lymphocytes et les monocytes, jouent un rôle crucial dans le développement de la lésion d'athérosclérose, et il existe un intérêt croissant pour comprendre comment les facteurs nutritionnels peuvent modifier l'activité de ces cellules en rapport avec le risque de maladies cardiovasculaires (13). Beaucoup d'études d'intervention

ont montré que les facteurs nutritionnels, tels que les acides gras insaturés et la vitamine E, peuvent moduler la fonction immunitaire de telle sorte que la progression de la maladie soit retardée. Même si dans des études in vitro il a été montré que des polyphénols pouvaient exercer des activités immunomodulatrices (14.15), on ne sait encore que peu de choses sur les effets physiologiques des polyphénols sur la fonction immunitaire lorsqu'ils sont consommés sous forme de produits végétaux frais ou transformés. La consommation en aigu de boissons riches en polyphénols, telles que le jus de raisins et le vin rouge dé-alcoolisé, n'a eu aucun effet sur le système immunitaire des hommes en bonne santé (16). En revanche, chez des sujets sains recevant pendant 2 semaines un régime pauvre en polyphénols supplémenté avec des jus de fruit riches en polyphénols (330 ml/j), apportant une variété de polyphénols à doses physiologiques, on observe des modifications de divers biomarqueurs du statut immunitaire se traduisant par une stimulation des fonctions immunitaires (17). D'ailleurs l'ingestion de doses physiologiques de polyphénols via la consommation de feuilles de patate douce (200g/j pendant 2 semaines) affecte favorablement le statut immunitaire des sujets sains (18).

Dans l'étude que nous mettons en place, nous comptons aussi prendre en considération l'impact des polyphénols des agrumes in vivo sur l'expression des gènes par une approche transcriptomique. D'une façon générale, parmi les gènes dont l'expression semble être modifiée par des polyphénols ou des extraits végétaux, on a identifié notamment des facteurs de transcription, des cytokines, des molécules d'adhésion, c'est-à-dire des gènes qui pourraient jouer un rôle potentiel dans le développement des maladies cardiovasculaires (19-21). En conséquence il est intéressant de comparer les profils d'expression des gènes obtenus chez des individus après consommation de jus d'orange ou de la boisson contrôle supplémentée ou non avec des polyphénols purifiés extraits de l'orange.

Les régimes occidentaux sont relativement riches en protéines et en sel et sont fréquemment considérés potentiellement acidogènes pouvant conduire à un déficit en bases alcalines. Ce déséquilibre acido-basique peut conduire à un état d'acidose métabolique latente à l'origine de divers troubles affectant les os, les reins ou les muscles avec des conséquences pathologiques majeures comme l'ostéoporose, la formation de calcul rénaux ou la sarcopénie (22.23). Le pouvoir alcalinisant du régime dépend de l'apport en potassium sous forme de sels d'anions organiques (24.25). Chez les consommateurs réguliers d'agrumes (fruits ou jus), la fourniture de ses anions organiques peut être en grande partie assurée par les agrumes puisqu'ils constituent une source majeure d'anions citrate.

Paraphe du volontaire avec la mention « lu et compris »

Les agrumes sont consommés sous forme de fruit frais ou le plus souvent sous forme de jus. Plusieurs facteurs relatifs à leur composition laissent penser qu'ils puissent être bénéfiques pour la santé. Ils sont riches en vitamine C et en phytomicronutrients (polyphénols et caroténoïdes), ils sont dépourvus de graisses, et de sodium. En outre ils contiennent des anions organiques, des minéraux (potassium, calcium, magnésium) et des folates. Par ailleurs, parmi les fruits, les agrumes sont des sources particulièrement intéressantes en polyphénols, puisqu'une portion de jus d'orange (200ml) contient entre 40 et 140 mg de flavanones (26). Ainsi, chez les forts consommateurs d'agrumes, l'apport en flavanones peut contribuer largement à la prise quotidienne de polyphénols totaux, estimée à environ 1 g/j. Une autre particularité intéressante des agrumes est qu'ils constituent l'unique source de flavanones de l'alimentation humaine. Les molécules de flavanones différent selon le type d'agrumes : l'hespérétine est la flavanone principale dans l'orange et la clémentine, la narigénine dans le pamplemousse et l'ériodyctiol dans le citron. Même si les flavanones d'agrumes sont à la fois parmi les polyphénols les plus consommés et ceux qui ont la meilleure biodisponibilité (9.27), leurs effets santé spécifiques ne sont pas encore entièrement établis et il est nécessaire de conduire des études cliniques. Les preuves cliniques du rôle d'une consommation d'agrumes dans la prévention des maladies cardiovasculaires concernent essentiellement leur effet hypocholestérolémique et l'amélioration de quelques biomarqueurs du stress oxydant (28.29). Cependant, la contribution spécifique des flavanones à ces effets n'a pas été examinée chez l'homme, mais seulement au travers d'études sur modèles animaux. Les agrumes (fruits et jus) peuvent aussi exercer des effets alcalinisant, favorables à la prévention de plusieurs maladies chroniques comme l'hypertension et l'ostéoporose (30). Même si le jus d'orange est perçu au goût comme une boisson acide, il est en fait capable d'exercer des effets alcalinisants après métabolisation du citrate de potassium.

Considérant l'ensemble de ces données bibliographiques, nous proposons dans le présent projet d'évaluer chez des volontaires sains d'âge moyen (50 à 65 ans) en léger surpoids et suivant leur régime habituel :

(1) les effets en prise unique et en prises répétées de l'absorption de jus d'orange ou de flavanones purifiées extraits d'oranges sur la fonction vasculaire, en mesurant la réactivité microvasculaire dépendante de l'endothélium et les biomarqueurs associés au risque cardiovasculaire.

Paraphe du volontaire avec la mention « lu et compris »

(2) les effets d'une consommation répétée de jus d'orange ou de flavanones purifiées sur la fonction immunitaire en mesurant des biomarqueurs spécifiques et sur l’expression des gènes par une approche transcriptomique à partir de cellules mononucléaires isolées du sang périphérique.

(3) l'impact d'une ingestion chronique de jus d'orange sur des biomarqueurs du statut acido-basique très lié à la santé osseuse, ceci en déterminant l'équilibre minéral et l'excrétion urinaire des cations (K, Ca, Mg) et des anions, en particulier citrate.

Paraphe du volontaire avec la mention « lu et compris »

2 Objectifs de l’étude

2.1 Objectif principal

L’objectif principal de la présente étude est d'évaluer, chez des volontaires sains d’âge mûr (50 à 65 ans) en léger surpoids et suivant leur régime habituel, les effets en prise unique (en période postprandiale) et en prises répétées de jus d'orange ou de flavanones d’orange purifiés sur la fonction vasculaire. Le choix des critères d’âge et de poids devrait permettre, à l’intérieur d’une population saine, de sélectionner des individus qui présentent des facteurs de risque cardiovasculaires, et donc pour lesquels il devrait être à priori plus facile de mettre en évidence un impact bénéfique de facteurs alimentaires sur les paramètres liés au risque cardiovasculaire.

2.2 Objectifs secondaires

Les objectifs secondaires sont au nombre de trois :

- Déterminer les effets d’une consommation répétée de jus d’orange ou de flavanones purifiés sur la fonction immunitaire
- Mesurer l’impact d’une consommation de jus d’orange ou de flavanones purifiés sur l’équilibre acido-basique
- Evaluer les effets chroniques d’une consommation de jus d’orange ou de flavanones purifiés sur l’expression des gènes par une approche transcriptomique globale dans les cellules mononuclées issues du sang périphérique.

3 Critères d’évaluation

Chez l’homme, la fonction vaso-motrice endothéliale est essentiellement évaluée par deux approches : la mesure de la Flow Mediated Dilation (FMD) réalisée sur l'artère brachiale ou bien la réactivité microvasculaire sous-cutanée mesurée par laser doppler après application d'acétylcholine ou de nitroprussiate de sodium par iontophorèse (31). Ces deux techniques non invasives présentent entre elles une bonne corrélation (32). En plus de ces mesures

fonctionnelles, il existe un certain nombre de biomarqueurs sanguins bien identifiés qui sont en rapport avec la fonction endothéliale chez l'homme (33). Une activation des cellules endothéliales stimule l'expression des chemokines et des molécules d'adhésion, ce qui conduit à un plus grand recrutement des cellules sanguines liées à l'inflammation au niveau de l'endothélium et à leur adhésion aux cellules endothéliales. L'activation par les lipoprotéines oxydées de faible densité (OxLDL) du récepteur LOX-1 (lectin-like oxidized LDL receptor-1) sur les cellules endothéliales induit (i) une régulation du MCP-1 (monocyte chemoattractant protein-1), de l'expression des molécules d'adhésion (ICAM-1 ; VCAM-1) et (ii) la réduction de la libération de NO (34). Tous ces évènements sont connus pour être à la base d'un dysfonctionnement endothélial. Le taux plasmatique du facteur de von Willebrand (vWF) est également un marqueur biochimique établi des dommages ou d'activation endothéliaux.

Il existe des données qui montrent que les polyphénols présents dans les aliments peuvent moduler la disponibilité de plusieurs de ces marqueurs endothéliaux, notamment celle du NO (35). L'oxyde nitrique est un puissant vaso-dilatateur et constitue une des molécules signal les plus répandues. A des niveaux physiologiques, le NO produit par les cellules endothéliales peut supprimer l'agrégation plaquettaire, exercer des propriétés anti-inflammatoires, influencer la production d'anion superoxyde, et atténuer la prolifération et la migration des cellules musculaires lisses (CML) (36).

La fonction endothéliale sera estimée, dans cet essai clinique, en mesurant la réactivité microvasculaire cutanée. Parallèlement, divers paramètres biochimiques liés au métabolisme des lipides, au stress oxydant et à l'inflammation seront également mesurés sur des échantillons de plasma prélevés à jeun et en période postprandiale.

3.1 Critère d'évaluation principal

L'administration transcutanée de substances vasodilatatrices par iontophorèse consiste à produire un champ électrique qui traverse une chambre de Perspex remplie de la solution contenant des formes ionisées (donc chargées) d'acétylcholine et de nitroprussiate de sodium. Ce champ électrique va faire migrer ces principes actifs de manière régulée en jouant sur l'intensité du courant électrique transcutané (qui est toujours inférieur à 1 mA). L'amplitude

de la charge iontophorétique (Q) est ainsi dépendante de la durée d'application (t) et de l'intensité du courant (I) délivré par l'anode ou la cathode selon les charges portées par les molécules ionisées. La charge ionique (Q exprimé en coulombs) est égale au produit de l'intensité (I exprimé en microampères) par le temps d'application (t exprimé en secondes).

Cette technique iontophorétique est utilisée chez l'homme depuis des décennies, y compris dans certaines pratiques de soins courants. Elle ne présente par elle-même aucun danger particulier.

Dans le cadre de ce protocole, les anneaux de feutrine situés sous l'anode ou la cathode elles-mêmes collées sur la peau seront imprégnés soit d'une solution à 2% d'acétylcholine (Sigma Chemicals LTD) diluée dans de l'eau déionisée ; soit une solution à 1% de nitroprussiate de sodium (Nipride®, Laboratoires Roche) diluée également dans de l'eau déionisée.

La réalisation de chaque test de réactivité microvasculaire sera conduite de manière identique tout au long du protocole d'étude.

Les sujets seront confortablement installés en position semi assise sur un fauteuil d'examen, dans une pièce calme dont la température sera stabilisée entre 23 et 25 °C.

Ils resteront ainsi au repos pendant au minimum 10 min. Pendant cette période, leur bras gauche sera confortablement positionné sur un accoudoir et la face interne de l'avant-bras sera délicatement dégraissée à l'aide d'un coton imprégné d'alcool à 60 °.

Un marquage du positionnement des électrodes utilisées pour la iontophorèse sera fait sur la face interne du bras. La cathode utilisée pour l'administration de l'acétylcholine sera positionnée à 10 cm du pli du poignet. L'anode destinée à l'administration du nitroprussiate de sodium sera placée à 17 cm du pli du poignet. La deuxième électrode reliée à l'appareil générateur de courant (PF 382 b Périlont Power Supply - PERIMED SA) sera placée sur la face externe du bras à 3cm du pli du coude.

Paraphe du volontaire avec la mention « lu et compris »

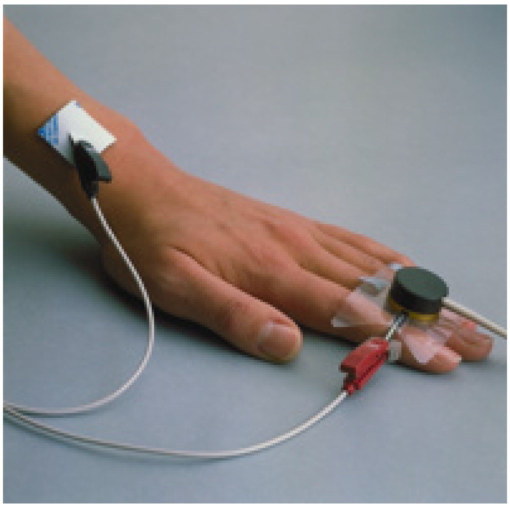

Type d'installation utilisée pour la mesure de la réactivité vasculaire par iontophorèse couplée au laser doppler (l'exemple de la photo montre un enregistrement sur la deuxième phalange du majeur a la place de la face interne de l'avant bras retenu dans ce protocole (Document PERIMED ©)

Dans un premier temps, on évaluera l'action de l'acétylcholine appliquée par iontophorèse au niveau de l'électrode la plus distale avec une augmentation par palier de la charge iontophorétique répondant au protocole suivant :

- enregistrement du débit capillaire basal sous la cathode pendant 120 sec
- premier palier : courant électrique à 40 µA pendant 10 sec (0,4 mC) puis interruption pendant 40 sec durant lesquelles on mesure la réponse vasodilatatrice à l'aide du laser Doppler
- deuxième palier : courant électrique à 100 µA pendant 10 sec (1 mC) puis interruption pendant 40 sec
- troisième palier : courant électrique à 200 µA pendant 10 sec (2 mC) puis interruption pendant 60 sec
- quatrième palier : courant électrique à 200 µA pendant 20 sec (4 mC) puis interruption pendant 90 sec
- cinquième palier : courant électrique à 200 µA pendant 40 sec (8 mC) puis interruption pendant 120 sec.

La durée totale de l'épreuve avec l'acétylcholine est de 520 secondes.

Paraphe du volontaire avec la mention « lu et compris »

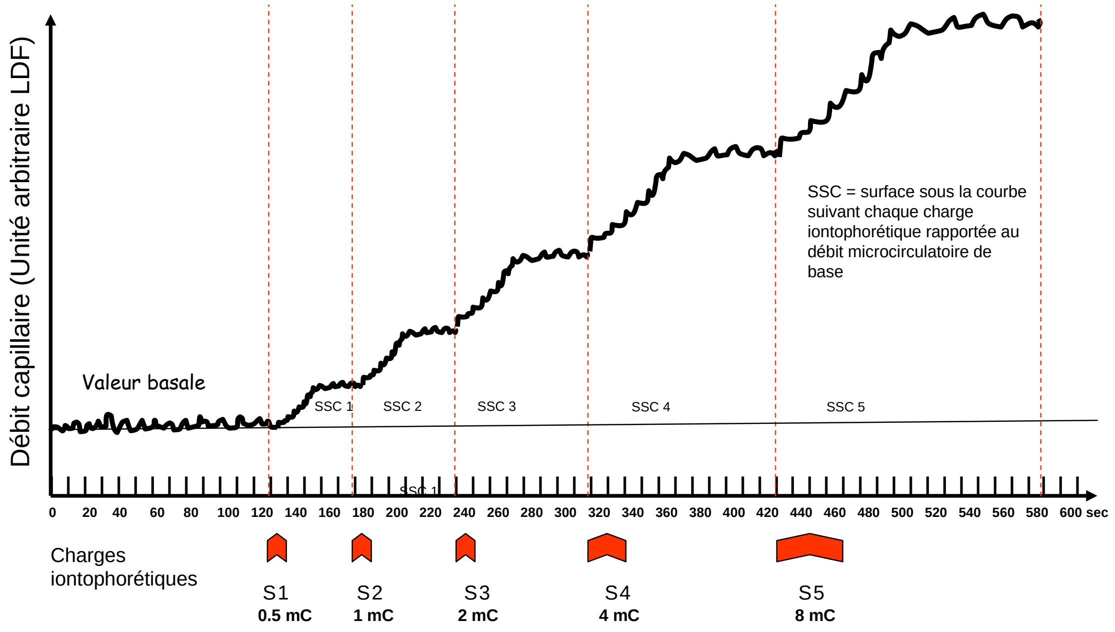

Durant un intervalle de 120 à 180 sec, on positionnera l'anode destinée à l'application locale du nitroprussiate de sodium.

On effectuera un enregistrement basal sous la cathode pendant 120 sec puis une progression sur 5 paliers identiques à ce qui a été décrit pour l'acétylcholine.

L'enregistrement du débit micro circulatoire sous-cutané dans chacune de ces épreuves sera réalisé à l'aide d'une sonde (PF 481). Cette sonde est percée d'un orifice situé au centre de l'électrode circulaire du système de iontophorétique. A travers l'orifice, la fibre dirigeant le faisceau laser mise au contact de la surface cutanée pour mesurer le débit capillaire instantané selon le principe des échos doppler (PERIFLUX – PERIMED SA). Les réponses vaso-dilatatrices seront enregistrées au cours de l'iontophorèse de l'acétylcholine pour mesurer la vasomotricité dépendante de l'endothélium et de l'iontophorèse du nitroprussiate de sodium, qui est une molécule donneuse de NO, pour mesurer la vasomotricité indépendante de la fonction endothéliale au niveau de l'avant-bras (31). Cette méthode a été validée et précédemment décrite en détail (37,38).

La réponse vasomotrice enregistrée par le PERIFLUX sera calculée à l'aide d'une mesure de la surface sous les courbes du débit capillaire cumulant les réponses des 5 paliers d'application des substances vasoactives.

Paraphe du volontaire avec la mention « lu et compris »

L’ensemble de ces 2 tests de vasoréactivité peut être réalisé en moins de 25 minutes.

Les temps auxquels la réactivité vasculaire sera mesurée ont été choisis afin de toujours respecter un délai de 30 minutes après une prise de sang. Compte tenu de la littérature et de ce que nous voulons montrer, les points de mesure pour les deux études cinétiques postprandiales (en aigue et en chronique) sont différents.

Les différents temps de mesure de la réactivité microvasculaire seront les suivants :

- *Dans l’étude en prise unique*, les mesures de réactivité vasculaire seront faites à jeun et à T+4,5 et T+6,5 heures après le traitement A, B ou C (T0 étant le temps auquel le sujet prend le traitement). La prise du traitement sera accompagnée d’un petit déjeuner sans polyphénols et équilibré en macronutriments. Les temps de prélèvements prennent en compte la cinétique d’absorption des flavanones, la concentration plasmatique maximale en flavanones étant atteinte au bout de 6heures. Pour chaque volontaire, les mêmes mesures, dans les mêmes conditions, seront effectuées à l'inclusion, c’est-à-dire une semaine avant le début du protocole pour déterminer les valeurs basales de la réactivité microvasculaire après la consommation d’eau plate.

- *Dans l'étude en prises répétées*, la réactivité vasculaire sera mesurée en conditions de jeûne à la fin de chaque traitement (4 semaines avec A, B ou C), et après la consommation d'un repas à forte teneur en graisses identique pour tous les volontaires, plus précisément à T+2,5 et T+4,5 heures après l'ingestion du repas test. La durée totale impartie pour consommer le repas est de 20 min, le T0 de la cinétique postprandiale correspond au milieu du repas (il se situe donc 10 min après le début de la prise du repas). Ces temps de mesure de la réactivité vasculaire ont été fixés en fonction des données de bibliographie (10).

Paraphe du volontaire avec la mention « lu et compris »

3.2 Critères d'évaluation secondaires

3.2.1 Détermination des biomarqueurs associés au risque cardiovasculaire

- Biomarqueurs de la fonction endothéliale

Les marqueurs solubles de la lésion endothéliale (facteur von Willebrand) et de l'activation endothéliale (molécules d'adhésion : sICAM-1 et sVCAM-1), seront déterminés par des techniques ELISA dans le plasma des sujets à jeun, au début et à la fin de chacune des 3 périodes expérimentales et aussi pour tous les prélèvements sanguins effectués après l'ingestion du repas riche en graisses. Le pool de NO circulant sera déterminé tout au long du protocole à chaque fois que des prélèvements sanguins seront effectués (39). Pour l'ensemble de ces mesures, les échantillons de sang seront collectés sur héparine ou sur EDTA.

- Biomarqueurs du stress oxydatif

La capacité antioxydante totale du plasma estimée en terme de FRAP (ferric reducing activity of plasma) sera utilisée en tant que biomarqueur de la protection antioxydante globale. Différents antioxydants comme la vitamine E, vitamine C, et l'acide urique seront mesurés dans le plasma. Ces mesures seront faites avant (à jeun) et en cinétique après la première prise de chaque traitement (étude postprandiale en prise unique) ainsi que sur les prélèvements sanguins réalisés à jeun à la fin de chaque période expérimentale.

- Paramètres biologiques liés aux facteurs de risque vasculaire

Le premier jour de chaque période expérimentale, les concentrations plasmatiques en triglycérides, cholestérol et glucose seront mesurées pour chaque prélèvement sanguin de la cinétique post prandiale. Dans l'étude en prises répétées, la glycémie et le profil des lipides sanguins (cholestérol total et triglycérides mesurés par spectrophotométrie en utilisant une méthode enzymatique), et HDL-cholestérol (mesuré directement par colorimétrie en utilisant le test enzymatique approprié (Randox)) seront déterminés.

Au début et à la fin de chaque période expérimentale, dans le sérum ou le plasma des sujets à jeun, les paramètres listés ci-dessus ainsi que les marqueurs d'inflammation systémique : CRP (par turbidimitrie) et IL-6 (par ELISA ; Labsystems) seront mesurés.

Paraphe du volontaire avec la mention « lu et compris »

Le dernier jour de chaque période expérimentale, après l'ingestion du repas test à forte teneur en graisses, les taux plasmatiques de glucose, de triglycérides, de cholestérol ainsi que ceux d'IL-6 seront mesurés pour chaque prélèvement sanguin (T+2h, +4h et +6h après l'ingestion).

3.2.2 Evaluation du phénotype des cellules immunitaires et de leurs fonctions

Nous évaluerons si la prise de doses physiologiques de flavanones d'agrumes via la consommation de jus d'orange ou de composés purifiés peut affecter le statut immunitaire de sujets sains.

- ***Phénotype des cellules immunocompétentes du sang***

La caractérisation des cellules immunocompétentes sera réalisée en cytométrie en flux en utilisant différentes combinaisons de fluorochromes conjugués à des anticorps. Ces mesures reposent sur la capacité d'anticorps monoclonaux spécifiques à se fixer sur les cellules leucocytaires par le biais des déterminants antigéniques qu'elles expriment. Brièvement, le marquage spécifique des cellules consiste à incuber le sang avec des anticorps et après lyse des érythrocytes, à analyser l'intensité de fluorescence par cytométrie en flux. L'intensité de fluorescence est proportionnelle à chaque type cellulaire détecté.

- ***Evaluation de la des cellules immunitaires.***

- Prolifération des lymphocytes, ce paramètre sera déterminé par la mesure du pourcentage de cellules exprimant à leur surface le récepteur à l'interleukin-2 (IL-2, % de cellules $CD25^{+}$) et de la densité réceptoriale.

- Production de cytokines par les cellules mononucléées et plus particulièrement évaluation de l'équilibre Th1-Th2. La balance entre la production de cytokines de type Th1 et Th2 conditionne la réponse immunitaire vers une réponse cellulaire ou humorale (40). Les cellules immunocompétentes de type Th1 produisent des cytokines pro-inflammatoires dont l'IL-2 et l'interféron (IFN-γ) qui activent les monocytes/macrophages, les cellules natural killer (NK) et les cellules T cytotoxiques. Elles sont principalement associées à l'immunité cellulaire. Les cellules de type Th2 produisent des cytokines anti-inflammatoires telles qu'IL-4, IL-5 et IL-13 et seraient associées préférentiellement à l'immunité humorale (41).

Brièvement, après culture (48h) des cellules mononuclées dans un milieu contenant ou non de la phytohémagglutine (PHA, 5µg/ml), les concentrations en cytokines (IL-2 et IL-4) dans les surnageants de culture seront mesurées par une technique ELISA selon les instructions du fabricant (kit ELISA, Beckman-Coulter).

- ***Activité lytique des cellules natural killer (NK)*** vis-à-vis des cellules cibles K562. Elle sera mesurée par cytométrie en flux. Les cellules NK sont les acteurs principaux de l'immunité innée puisqu'elles possèdent la capacité de développer une cytotoxicité spontanée sans infection antérieure et ainsi semblent participer directement ou indirectement à l'homéostasie du système immunitaire (42). Une augmentation de l'activité lytique des cellules de NK consécutive à une exposition à des composés spécifiques reflète *in vitro* l'activité immunosuppressive de ces derniers.

Brièvement, les cellules NK sont incubées avec des cellules cibles (K562-EGFP), lignée de cellules érythroleucémiques humaines exprimant de façon stable la protéine EGFP (Enhanced Green Fluorescent Protein). La cytotoxicité des cellules NK est déterminée par le pourcentage de cellules (K562-EGFP) lysées par les cellules NK.

- ***Production d'espèces oxygénées réactives (EROs) par les monocytes et polynucléaires neutrophiles.*** Ces cellules sont impliquées dans la première ligne de défense de l'hôte. Une fois activées, elles produisent des espèces réactives de l'oxygène. En effet, elles possèdent le complexe NADPH/H+ oxydase qui catalyse la conversion de l'oxygène moléculaire en anion superoxyde, ce dernier étant ensuite rapidement converti en peroxyde d'hydrogène (H_2O_2) (43). Brièvement, les cellules immunocompétentes isolées du sang seront mises en contact avec une sonde intracellulaire (dihydrorhodamine 123) qui a pour propriété de devenir fluorescente lorsqu'elle est oxydée en rhodamine 123 en présence d' H_2O_2. Ce processus d'oxydation est induit par le phorbol-12-myristate-13-acétate (PMA à 10^{-6}M) qui stimule directement la NADPH oxydase. L'augmentation de la production de radicaux libres oxygénés se traduit par une élévation de l'oxydation de la DHR et donc par une augmentation de l'intensité de fluorescence qui est quantifiée par cytométrie en flux.

Les divers paramètres liés à l'immunité cités ci-dessus seront mesurés chez les sujets à jeun au début et à la fin de chaque période expérimentale. Pour cela, 20 ml de sang périphérique veineux seront prélevés dans des tubes vaccutainer contenant de l'EDTA au début et à la fin de chaque période expérimentale.

Paraphe du volontaire avec la mention « lu et compris »

3.2.3 Evaluation de l’équilibre acido-basique

Nous rechercherons si la consommation régulière de jus d’orange dans les conditions fixée par le protocole (500ml/j pendant 4 semaine) : (1) modifie de façon substantielle l'excrétion urinaire de K, de Ca et de Mg, et (2) provoque des changements spécifiques du profil anionique dans les urines et une augmentation de la citraturie, ce qui serait le reflet d’une alcalinisation.

Seuls les conditions expérimentales A et B seront prises en compte pour cette partie de l’étude. Cependant, étant donné que l’étude se déroule en aveugle pour les périodes B et C, les prélèvements nécessaires aux mesures seront effectués pour l’ensemble des périodes.

En fin de chaque période expérimentale, la veille du dernier jour (J+27) les volontaires devront recueillir leurs urines depuis le matin au réveil, jusqu’au lendemain matin au réveil et ils amèneront leurs urines de 24h au CIC le matin de J28 (jour de visite). Les flacons nécessaires à ces recueils seront fournis aux volontaires à l’occasion des visites au CIC prévues en début de période.

Le pH urinaire sera estimé en utilisant des bandelettes de papier indicatrices de pH. La détermination des taux urinaires de K, Ca et Mg sera effectuée par spectrométrie d’absorption atomique, le Na par photométrie de flamme et l'ammonium, l'urée et la créatinine en utilisant des kits spécifiques. Les taux urinaires de chlorure, sulfate, oxalate, phosphate et citrate seront déterminés par chromatographie ionique (Dionex).

3.2.4 Analyse transcriptomique des cellules PBMC isolées

L’impact de la consommation de jus d'orange ou de flavanones purifiées sur l’expression des gènes des cellules mononuclées issues du sang périphérique (Peripheral Blood Mononuclear Cells : PBMCs) sera caractérisée en utilisant une approche transcriptomique. Les PBMC peuvent être facilement isolées et en quantités suffisantes pour permettre l’extraction d'ARN afin de mesurer l'expression des 25.000 gènes du génome humain (ce qui permet d’obtenir un profil global de l’expression des gènes) par la technique des microarrays (lames de verres contenant des oligonucléotides de 50 à 55 pb des 25.000 gènes). L’utilisation des cellules sanguines présente également un avantage supplémentaire :

le sang constitue un milieu fortement dynamique, communiquant avec pratiquement chaque tissu de l’organisme et à ce titre peut être considéré comme un "tissu sentinelle" répondant aux différentes conditions environnementales (44).

L’approche génomique est employée pour distinguer des individus selon leur état physiologique ou de santé, et pour caractériser la réponse à des divers facteurs environnementaux. Par exemple, des études cliniques ont montré que l'expression des gènes est modifiée dans les PBMCs suite à l'exposition au benzène et après des exercices physiques intenses (45). Les profils d’expression des gènes des cellules PBMCs ont aussi été utilisés pour distinguer des individus présentant ou non un carcinome rénal (46). Par ailleurs, récemment il a été observé que la consommation de petits déjeuners isoenergétiques mais riches en macronutriments différents (glucides ou protéines) s’accompagnait de changements importants dans le profil d’expression des gènes des cellules sanguines (48). Des modifications d'expression des gènes codant pour des cytokines ont aussi été décrites dans les cultures de PBMCs exposées à des polyphénols tels que des procyanidines isolées de cacao (47). En utilisant cette approche transcriptomique, il est possible d'analyser les niveaux d'expression des milliers de gènes par une analyse simple, permettant d’examiner simultanément de nombreux voies moléculaires et donc de faciliter l’identification des mécanismes moléculaires spécifiquement modifiés par l'ingestion du jus d'orange ou des flavanones purifiés.

Analyse transcriptomique : pour chaque volontaire à la fin de chaque période expérimentale, 20 ml de sang seront prélevés sur EDTA. La moitié des volontaires sera prélevée à jeun, tandis que l’autre moitié le sera 2 heures après la prise du repas riche en graisses. Ces deux groupes de sujets seront définis dès le début de l’étude (au moment de la randomisation de la première période) et seront identiques tout le long de l’étude. Les PBMC seront immédiatement isolés à partir du sang total en utilisant les kits commerciaux adaptés et les ARN totaux extraits à partir des cellules obtenues. Les ARN, obtenus à partir des cellules PBMC des sujets ayant reçu le régime A ou C, seront rétro-transcrits et marqués en fluorescences (cyanins 3 et 5) puis hybridés sur les microarrays avec des ADNc marqués des sujets qui ont reçu le régime contrôle (régime B). Les microarrays qui seront employés dans cette étude contiendront 25.000 oligonucléotides correspondant à environ 23.000 gènes différents et seront acquis du réseau national des génopôles (RNG). Des hybridations

réciproques (dye-swap) seront systématiquement effectuées. Les microarrays seront scannés et analysés par le logiciel : Imagene (BioDiscovery) avant des analyses statistiques des données afin d’identifier des gènes présentant une expression différentielle. Une validation des résultats sera effectuée par PCR en temps réel. Les études bioinformatiques seront réalisées sur les gènes différentiellement exprimés afin d'identifier des voies cellulaires modifiées par des différents régimes.

4 Méthodologie de la recherche

4.1 Déroulement de l’étude

24 volontaires participeront à l’étude randomisée, ouverte, contrôlée et en cross-over. Chaque sujet suivra successivement et pendant 4 semaines chacun trois traitements alimentaires qui consisteront en :

A) 500 ml de jus d'orange

B) 500 ml d'une boisson contrôle contenant 50g de sucre (correspondant à la quantité de sucre apportée dans le jus d'orange)

C) 500 ml de la boisson contrôle supplémentée avec X mg d’hespéridine (X= quantité d’hespéridine (en mg) apportée par 500 ml de jus d’orange).

La quantité de sucres présents dans la boisson contrôle (groupes B et C) sont identiques à celles du jus d'orange. Dans ces conditions, les trois traitements sont isocaloriques (environ 200 Kcal).

Le premier jour de chaque période (J0), les volontaires prendront le traitement en une seule prise, le matin à jeun au CIC. Par contre pour le reste de chaque période (J+1 à J+27), les doses quotidiennes (boisson et supplément) seront réparties en 2 prises équivalentes : la première moitié sera ingérée au petit déjeuner, la seconde au moment du déjeuner.

Dans le groupe A, le jus d’orange utilisé sera celui fournit par la Fédération des producteurs de Floride. Le jus d’orange pasteurisé sera distribué aux volontaires sous la forme de bouteilles de 1litre. Pour couvrir la période de l’étude ou les volontaires devront

consommer le jus d'orange chez eux (entre J+2 et J+27), cela impliquera de leur distribuer 15 litres de jus (13litres nécessaires pour l'étude + 2litres en réserve) qu'ils devront conserver à l'abri de la lumière jusqu'à leur utilisation. En même temps que les bouteilles de jus d'orange, il sera également remis aux volontaires en début de période lors de leur visite au CIC, un verre de 250 ml qui facilitera la prise quotidienne de 2x 250 ml de boisson.

Dans les groupes B et C, à chaque prise les volontaires devront prendre un sachet de sucre et une gélule :

- La prise du sachet de sucre apportera 25g de saccharose, soit la moitié de la dose quotidienne. Le sucre sera mis en suspension dans 250 ml d'eau par les volontaires avant chaque prise.

- dans le groupe C, chaque gélule contiendra Xmg/2 d'hespéridine, avec X= quantité d'hespéridine (en mg) apportée par 500 ml de jus d'orange. Préalablement au démarrage de l'étude, la teneur en flavanones dans le jus d'orange utilisé dans le groupe A aura été analysée par HPLC à l'Unité de Nutrition Humaine de l'INRA de Theix. Cette analyse permettra de connaître X (mg), correspondant à l'apport quotidien en flavanone via la consommation de 500 ml de jus d'orange. L'hespéridine purifiée extraite à partir des peaux d'orange et de mandarine, sera achetée auprès de la société Nutrafur (Murcia, Espagne) et distribuée aux volontaires sous forme de gélules.

- Dans le groupe B, chaque gélule contiendra une poudre placebo (lactose).

Les 2 types de gélules seront conditionnés dans des piluliers.

Le conditionnement de l'hespéridine et du placebo en gélules sera réalisé par la pharmacie du CHU Gabriel Montpied.

En début de chaque période (B ou C), il sera remis aux volontaires le jour de leur visite au CIC, un coffret contenant la totalité des produits à consommer (sucre en sachet + gélules).

Chaque traitement (A, B, C) sera associé au régime habituel des volontaires. Pendant le protocole, la consommation totale de sucre sera contrôlée et toute consommation supplémentaire d'agrumes ou de produits dérivés (jus, confitures...) sera interdite.

Les volontaires suivront les 3 traitements alimentaires pendant 4 semaines et les périodes expérimentales seront séparées les unes des autres par une période de 3 semaines. Un tiers des sujets commenceront par le traitement A, un tiers avec le traitement B et un tiers avec le traitement C.

Paraphe du volontaire avec la mention « lu et compris »

Chaque volontaire se rendra au Centre d'Investigation Clinique (CIC) le premier et dernier jour de chaque période d'intervention (voir figure 1 en annexe 1). Les effets en prise unique et en prises répétées de chacun des 3 traitements seront évalués comme suit :

- ***Les effets vasculaires en prise unique*** consécutifs à la prise du jus d'orange ou d'une supplémentation en flavanones purifiés seront étudiés en cinétique le premier jour de chaque période de traitement. Au CIC (Visite 2, 4 et 6), le matin à jeun, les volontaires recevront en une seule prise la totalité de la dose quotidienne de chaque traitement A, B ou C (correspondant à un apport de l'ordre de 200 Kcal), accompagnée d'un petit déjeuner composé de : ¼ baguette + 10 g beurre + 50g jambon (soit 279 Kcal). En cours de journée, ils ne consommeront rien d'autre (sauf de l'eau) jusqu'à la fin des mesures de réactivité vasculaire. Avant de partir du CIC, les volontaires prendront sur place une collation puis ils rentreront chez eux.

Tout au long de la journée, des mesures de réactivité vasculaire (à jeun, et 4,5 et 6,5 heures après la prise) et des prélèvements sanguins (à jeun et 2, 4, 6 heures après la prise du traitement) seront réalisés. La liste des paramètres qui seront analysés sur ces échantillons de plasma est présentée dans la figure 2 (annexe 2). A jeun, ce sont 15mL de sang qui seront prélevés et à chaque point de mesure en cinétique (T+2, +4 et +6 heures après la prise du traitement), 12 mL de sang seront prélevés. Au total, à chacune des visites au CIC en début de chaque période expérimentale (V2, V4 et V6), ce sont 51ml de sang qui seront prélevés pour évaluer les effets vasculaires.

D'autre part, pour chaque volontaire une semaine avant le début du protocole (V1, visite d'inclusion) (voir figure 1 en annexe 1), les mêmes mesures de réactivité et prélèvements sanguins seront effectués aux mêmes temps que lors des visites V2, V4 et V6 pour déterminer les valeurs basales de différents paramètres (listés sur la figure 2 en annexe 2) après la consommation de 500 ml d'eau du robinet (+ petit déjeuner décrit précédemment). Pour cela ce sont 4 x 12 ml de sang (soit 48ml) qui seront prélevés lors de la visite d'inclusion (V1) des volontaires au CIC.

Paraphe du volontaire avec la mention « lu et compris »

- ***Les effets en prises répétées*** consécutifs à la consommation de jus d'orange ou de supplément de flavanones purifiés seront déterminés après la prise quotidienne (répartie en 2 fois) de chaque traitement pendant 4 semaines :

o *en conditions de jeûne* : l'impact au niveau vasculaire sera déterminé en suivant l'évolution de la réactivité vasculaire et des biomarqueurs associés au risque cardiovasculaire entre le début et la fin de chaque période expérimentale. Pour ce volet vasculaire, la mesure des paramètres sanguins nécessitera de prélever 15 ml de sang en début et en fin de chaque période. L'exploration de la fonction immunitaire sera réalisée à partir de prélèvements de sang (20 ml) effectués à jeun en début et en fin de période. A la fin de chaque période expérimentale, 20 ml de sang seront aussi prélevés pour l'analyse transcriptomique sur PBMC sur la moitié des volontaires. Finalement en condition de jeune, sur la totalité d'une période, ce sont 90 ml de sang qui devront être prélevés sur la moitié des volontaires pour réaliser tous les volets de l'étude, et seulement 70 ml pour l'autre moitié pour laquelle l'étude transcriptomique ne sera pas réalisée dans les conditions de jeûne.

o *En réponse à un stress métabolique postprandial induit par la consommation d'un repas riche en graisses* : le dernier jour de chaque période (J+28), les volontaires se rendront à jeun au CIC et consommeront tous sur place un repas riche en graisse de composition définie. L'ingestion d'un tel repas a pour conséquence l'induction d'un stress postprandial connu pour modifier défavorablement la réactivité vasculaire et les paramètres du stress oxydant (10). Le repas test sera constitué de : 1/3 de baguette, 30g de beurre et 180g d'emmental, apportant 78g de lipides, 49g de glucides et 55,4g de protéines (soit 1135 Kcal). La composition de ce repas a été élaboré en fonction de l'étude de Schinkovitz et al, 2001 qui a étudié l'influence d'un repas riche en graisses sur la réactivité vasculaire et sur les biomarqueurs du stress oxydant chez des volontaires sains (49). La prise de ce repas sera accompagnée d'eau à volonté et il n'y aura pas de consommation d'aucune boisson test ce jour là. Le temps imparti pour la consommation du repas sera de 20 minutes. Le T0 sera donné au bout de 10 minutes, c'est-à-dire au milieu de la prise du repas.

La réactivité vasculaire cutanée (à jeun, 2,5 et 4,5 heures après la prise du repas test) sera mesurée et des prélèvements sanguins seront réalisés (à jeun et 2, 4, 6 heures après la prise du repas test). Les mesures qui seront faites sur ces échantillons de plasma sont énumérées dans la figure 3 (annexe 3). Le prélèvement de sang à jeun sera le même que celui

cité dans le paragraphe précèdent « *en conditions de jeûne* ». A chaque point de la cinétique 5 mL de sang seront prélevés pour les aspects vasculaires, ainsi que 20 ml au point T+2h sur la moitié des volontaires pour l'étude transcriptomique.

° *Evaluation de l'équilibre acido-basique au bout de 4 semaines de traitement* : les mesures relatives à cette étude seront réalisées à la fin de chaque période sur des recueils d'urine de 24h qui seront effectués par les volontaires entre les matins au réveil de J+27 et J+28. Pour effectuer ces recueils les volontaires disposeront de flacons qui leur auront été remis à chaque début de période expérimentale (J0) lors de leur visite au CIC. Les recueils d'urine seront déposés au CIC par les volontaires le matin de J+28, lors de la visite de fin de période. Même si seules les conditions expérimentales A et B seront prises en compte pour cette partie de l'étude, l'administration des traitements B et C étant réalisés en aveugle, toutes les urines seront récoltées en fin de période.

Bilan des prélèvements sanguins effectués au CIC tout au long de l'étude :

- **Visite de sélection** : 13 mL pour la détermination des paramètres biologiques liés à l'inclusion (7mL sur tube jaune sur gel de 7 mL pour les sérologies VIH et Hépatite C, 1 tube violet de 2mL pour la NFS et 1 tube vert de 4 mL pour les analyses suivantes : ASAT, ALAT, Phosphatase Alcaline, GGT, Créatinine, urée, glucose, TG, cholestérol total, HDL cholestérol, LDL cholestérol et CRP.

- **Visite 1** (inclusion): pour la détermination de l'évolution des taux de bases en cinétique pour le volet vasculaire après consommation d'eau, à J-8 : (4x12 ml) 48 ml de sang (4x9mL sur Héparine/ EDTA et 4x3mL sur Héparine seule)

- **Au cours des visites V2, V4 et V6 effectuées en début de chaque période** (à J0) il sera prélevé à chaque fois:

  - A jeun, 35 ml : 15 ml pour le volet vasculaire (10 mL sur Héparine EDTA et 5 mL sur Héparine seule) + 20 ml sur Héparine/ EDTA pour la fonction immunitaire
  - Pour chaque cinétique post prandiale (T+2h, T+4h, T+6h): 36 ml (3x12mL) pour le volet vasculaire (3x9mL sur Héparine/ EDTA et 3x3mL sur Héparine seule)

Paraphe du volontaire avec la mention « lu et compris »

- **Au cours des visites V3. V5 et V7 effectuées en fin de chaque période** (à J+28), il sera prélevé à chaque fois :

- A jeun :

- pour la moitié des sujets : 55 ml : 15 ml pour le volet vasculaire (10 mL sur Héparine EDTA et 5 mL sur Héparine seule) + 20 ml sur Héparine/ EDTA pour la fonction immunitaire + 20 ml sur Héparine/ EDTA pour l'étude transcriptomique.

- pour l'autre moitié des sujets : 35 ml : 15 ml pour le volet vasculaire (10 mL sur Héparine EDTA et 5 mL sur Héparine seule) + 20 ml sur Héparine/ EDTA pour la fonction immunitaire.

- En cinétique post prandiale après induction d'un stress lipidique (à T+2h, T+4h, T+6h) :

- pour la moitié des sujets : 3x5 ml sur Héparine/ EDTA pour le volet vasculaire

- pour l'autre moitié des sujets : 3x5 ml sur Héparine/ EDTA pour le volet vasculaire + 20 ml sur Héparine/ EDTA pour l'étude transcriptomique

Finalement entre le début et la fin de chaque période expérimentale, ce sont 141 ml de sang qui seront prélevés. Pour les 3 périodes expérimentales, cela représente 423 ml de sang.
En prenant en compte les volumes de sang prélevés lors de l'inclusion (13 mL) et ceux de la visite n°1 (48 ml), ce sont 484 ml de sang qui seront prélevés par volontaires pour l'ensemble de l'étude.

Bilan des recueils urinaires effectués au CIC tout au long de l'étude :

- A la fin de chaque période expérimentale, les urines seront recueillies sur 24h : entre le matin au réveil de J+27 et le matin au réveil de J+28. Ces recueils serviront à l'étude de l'équilibre acido-basique et aux vérifications de compliance (voir section 4.5).

4.2 Recrutement des volontaires

Paraphe du volontaire avec la mention « lu et compris »

L'étude sera conduite, selon les recommandations du guide des bonnes pratiques cliniques, au Centre d'Investigation Clinique (CIC) (CHU, Clermont-Ferrand) qui est sous-traitant dans le projet. Le protocole sera soumis à l'approbation du comité d'éthique (CPP). Les volontaires seront recrutés d'après une liste de volontaires disponible au CIC et par la publicité qui sera faîte dans les journaux locaux. Pendant quatre à six semaines avant que l'étude ne débute, le CIC évaluera si tous les volontaires sont en bonne santé sur la base d'un examen clinique approprié.

L'examen inclura un questionnaire de santé et de style de vie, un examen par un médecin, les recherches des sérologies VIH et hépatite C, la mesure de tension artérielle, les analyses sanguines suivantes : Numération Formule Sanguine, ALAT/ASAT, phosphatases alcalines, γGT, créatinine, urée, glucose, lipides (triglycérides, cholestérol total, HDL-chol, LDL-chol), protéine C-réactive (CRP), seront aussi déterminés. Suite aux analyses, les volontaires seront reçus par un médecin qui informera confidentiellement chaque volontaire des résultats de l'examen médical. Chaque volontaire répondant aux critères de recrutement se verra expliqué le protocole dans le détail et sera invité à donner son consentement par écrit pour sa participation (document en annexe 4). Il lui sera versé une indemnité de 1130 euros pour accomplir la totalité du protocole, à la fin de l'étude.

Les volontaires seront également invités à suivre leur alimentation habituelle tout au long de la période d'étude. L'étude inclura un suivi diététique (questionnaire de fréquence alimentaire) par une diététicienne afin d'évaluer, avant l'inclusion les habitudes alimentaires des volontaires puis pour détecter toute éventuelle modification de comportement alimentaire en cours d'étude.

4.3 Procédure de randomisation des sujets

La randomisation des sujets participant à l'étude se fera par un Attaché de Recherche Clinique du Centre d'Investigation Clinique. Celle-ci s'effectuera selon un carré latin permettant de constituer les six combinaisons possibles (ABC, BCA, CAB, ACB, CBA et

BAC). Ces six groupes seront équilibrés (4 sujets par groupe). Des enveloppes individuelles par sujet et par période de traitement seront préparées à l'intérieur desquelles l'investigateur découvrira quel traitement le patient doit recevoir.

S'il s'agit du traitement A (jus d'orange), l'investigateur et le sujet connaîtront la nature du traitement. Par contre, dans le cas du traitement C et B (sachet de sucre et gélule de flavanones purifiés d'une part, sachet de sucre et gélule placebo d'autre part), ni le sujet ni l'investigateur ne sauront la nature du contenu de la gélule.

4.4 Durée de participation des sujets de l'étude

Pour chaque volontaire, la durée totale de l'étude d'intervention sera de 19 semaines (voir figure 1, annexe 1).

4.5 Procédure de la vérification de la compliance des sujets

La compliance alimentaire sera évaluée comme suit : premièrement, les sujets seront invités à noter quotidiennement leur consommation en produits fournis (jus d'orange, sucres, gélules de flavanones ou de placebo) dans un carnet qui leur sera remis au début de l'étude. Ils seront également invités à ramener au CIC tous les produits non consommés à la fon de chaque période de l'étude. Deuxièmement, les taux de flavanones seront estimés par LC-MS sur les recueils d'urine de 24h réalisés entre les matins au réveil de J+27 et J+28 qui seront déposés au CIC par les volontaires à la fin de chaque période.

4.6 Données à recueillir lors de différentes visites

Les données recueillies lors de cette étude seront anonymisées. Un code sujet sera attribué à chaque sujet participant à l'étude. Seuls les investigateurs auront accès à la liste de correspondance entre le nom du sujet et son code.

4.6.1 Données à recueillir lors de la visite de sélection :

Lors de la visite de sélection, les données suivantes seront recherchées :

Paraphe du volontaire avec la mention « lu et compris »

- Consentement écrit
- Examen clinique et prélèvements sanguins permettant de vérifier les critères d'inclusion et de non-inclusion
- Mesure de la tension artérielle
- Réponse au questionnaire de santé et de style de vie
- Réponse au questionnaire de fréquence alimentaire

4.6.2 Données à recueillir lors de la visite d'inclusion (V1)

Détermination des valeurs basales (consommation d'eau) en cinétique pour tous les paramètres :

A jeun :

- mesure de réactivité microvasculaire (MRV1) : elle correspond à l'augmentation du débit capillaire enregistrée à l'aide d'un laser doppler en regard de l'électrode de iontophorèse sous l'influence de l'acétylcholine ou du nitropussiate de sodium. Cette augmentation est mesurée par comparaison des aires sous la courbe du débit capillaire à chaque palier d'intensité de courant.
- Concentrations plasmatiques de : triglycérides, cholestérol, glucose, FRAP, Vitamine C, acide urique, NO.

A T+2h : concentrations plasmatiques de triglycérides, cholestérol, glucose, FRAP, vitamine C, acide urique, NO

A T+4h : concentrations plasmatiques de triglycérides, cholestérol, glucose, FRAP, vitamine C, acide urique, NO

A T+4,5h : mesure de réactivité microvasculaire (MRV2)

A T+6h : concentrations plasmatiques de triglycérides, cholestérol, glucose, FRAP, vitamine C, acide urique, NO,

Paraphe du volontaire avec la mention « lu et compris »

A T+6,5h : mesure de la réactivité microvasculaire (MRV3)

4.6.3 Données à recueillir lors des visites 2, 4 et 6 (début de période)

A l'arrivée du sujet sur le lieu de la recherche, celui-ci remplira un questionnaire de fréquence alimentaire.

A jeun :

- mesure de réactivité microvasculaire (MRV1) : elle correspond à l'augmentation du débit capillaire enregistrée à l'aide d'un laser doppler en regard de l'électrode de iontophorèse sous l'influence de l'acétylcholine ou du nitropussiate de sodium. Cette augmentation est mesurée par comparaison des aires sous la courbe du débit capillaire à chaque palier d'intensité de courant.
- Concentrations plasmatiques de : triglycérides, cholestérol, glucose, FRAP, Vitamine C et E, acide urique, NO, HDL cholestérol, CRP, IL-6, vWF, ICAM, VCAM, flavanones, étude de la fonction immunitaire

A T+2h : concentrations plasmatiques de : triglycérides, cholestérol, glucose, FRAP, vitamine C, acide urique, NO

A T+4h : concentrations plasmatiques de : triglycérides, cholestérol, glucose, FRAP, vitamine C, acide urique, NO

A T+4,5h : mesure de réactivité microvasculaire (MRV2)

A T+6h : concentrations plasmatiques de : triglycérides, cholestérol, glucose, FRAP, vitamine C, acide urique, NO, flavanones

A T+6,5h : mesure de la réactivité microvasculaire (MRV3)

Paraphe du volontaire avec la mention « lu et compris »

4.6.4 Données à recueillir lors des visites 3, 5 et 7 (fin de période)

A l'arrivée du sujet sur le lieu de la recherche, celui-ci

- déposera le flacon du recueil d'urine de 24 h (collecte entre le matin au réveil de J+27 et le matin au réveil de J+28).
- remplira un questionnaire de fréquence alimentaire.

A jeun :

- mesure de réactivité microvasculaire (MRV1)
- concentrations plasmatiques de : triglycérides, cholestérol, HDL cholestérol, glucose, CRP, IL-6, FRAP, Vitamine C et E, acide urique, NO, vWF, I-CAM, V-CAM, étude de la fonction immunitaire, analyse transcriptomique pour la moitié des sujets.

A T+2h : concentrations plasmatiques de : triglycérides, cholestérol, glucose, NO, IL-6, vWF, ICAM-1, VCAM-1, analyse transcriptomique pour la seconde moitié des sujets

A T+2,5h : mesure de réactivité microvasculaire (MRV2)

A T+4h : concentrations plasmatiques de triglycérides, cholestérol, glucose, NO, IL-6, vWF, ICAM-1, VCAM-1,

A T+4,5h : mesure de la réactivité microvasculaire (MRV3)

A T+6h : concentrations plasmatiques de triglycérides, cholestérol, glucose, NO, IL-6, vWF, ICAM-1, VCAM-1,

Paraphe du volontaire avec la mention « lu et compris »

5 Description de la population de l'étude

5.1 Critères d'inclusion

- Sujet de sexe masculin
- Age : 50 à 65 ans inclus
- Indice de masse corporelle de 25 à 30 kg/m² (bornes incluses)
- Bilan biologique d'inclusion normal ou considéré comme normal par l'investigateur
- Ayant donné leur consentement écrit pour participer à cette étude

5.2 Critères de non-inclusion

- Hypertriglycéridémie ou hypercholestérolémie connues, traitées ou non
- Sérologie VIH et/ou VHC positive
- Don de sang dans les 3 mois précédant le début du protocole
- Diabète de type 1 ou type 2 (glycémie à jeun >7mmol/L) ou traitement antidiabétique
- Hypertension artérielle stade III (PAS>18 et/ou PAD>110)
- Pathologies de cancer, de maladie mentale ou toute autre pathologie sévère pouvant selon le médecin investigateur retentir sur le consentement éclairé et/ou sur les résultats obtenus.
- Antécédents médicaux et chirurgicaux lourds
- Antécédents de chirurgie digestive (sauf appendicectomie)
- Intervention chirurgicale récente (moins de 3 mois)
- Traitement médicamenteux dans les 2 mois précédents le protocole risquant d'interférer avec les résultats de l'étude : affectant la lipémie et la digestion (évalué par le médecin investigateur), principalement la prise de médicaments hypocholestérolémiants
- Consommation de tabac dans les six mois précédent la sélection du patient
- Alcoolisme (pas plus de 20g d'alcool par jour, soit 2 verres/j)
- Activité sportive importante (> 5h/semaine)
- Personne sous tutelle ou non assujettie à un régime de sécurité sociale
- Refus d'être inscrit sur le Fichier National des Volontaires

Paraphe du volontaire avec la mention « lu et compris »

- Personne en exclusion sur le Fichier National des Volontaires
- Personne en période d'exclusion d'une autre étude ou risquant de dépasser le plafond des indemnités annuelles pour la participation à des études cliniques

5.3 Critères d'ordre nutritionnels supplémentaires pour l'inclusion

- Pas de végétariens ou végétaliens
- Faible consommation de boissons riches en polyphénols (thé, café, vin, cacao, lait de soja, jus de fruits)
- Aucune consommation des suppléments en minéraux, vitamines ou polyphénols dans les 3 mois précédents le protocole.
- Pas d'aversion pour le fromage

6 Résumé des bénéfices et des risques prévisibles pour les volontaires

Les inconvénients pouvant résulter des prélèvements sanguins sont d'éventuels problèmes infectieux ou inflammatoires. Cependant ces complications sont exceptionnelles et toutes les mesures d'asepsie nécessaires seront prises pour les éviter. Il n'existe aucun inconvénient pouvant résulter de la consommation des trois traitements alimentaires (boisson et gélules). Il n'y à priori aucun inconvénient à consommer quotidiennement la dose de polyphénols d'orange contenue dans la gélule dans la mesure ou l'apport est nutritionnel puisque équivalent à celui apporté par 500 ml de jus d'orange.

Les données de la littérature rapportent de nombreux effets bénéfiques pour la santé associés à une consommation régulière et significative de produits d'origine végétale (légumes, fruits, boissons dérivées), naturellement riches en micronutriments (vitamines, caroténoïdes et polyphénols). L'apport en micronutriments devant être largement impliqué dans cette protection. Dans la présente étude on s'attend à une amélioration des paramètres systémiques et fonctionnels liés à la fonction vasculaire dans les groupes consommant le jus

d’orange ou des polyphénols d’orange purifiés en quantité équivalente à l’apport via le jus d’orange.

7 Procédures de déclaration des événements indésirables graves

L'article L209-12 du livre Il bis du Code de la Santé Publique fait obligation au promoteur d'une recherche biomédicale d'informer "dès qu'il en a connaissance, l'autorité administrative compétente de tout effet ayant pu contribuer à la survenue d'un décès, provoquer une hospitalisation ou entraîner des séquelles organiques ou fonctionnelles durables et susceptible d'être dû à la recherche". En outre, il doit transmettre "toute information relative à un fait nouveau concernant le déroulement de la recherche lorsque ce fait nouveau est susceptible de porter atteinte à la sécurité des personnes qui se prêtent à la recherche".

Cette déclaration implique tout événement indésirable grave.

Est qualifié d’indésirable toute manifestation nocive et non recherchée, subie par une personne participant à une recherche biomédicale, quelle que soit la cause de cette manifestation.

Est qualifié de grave tout événement dont l'évolution est fatale, ou qui est susceptible de mettre la vie en danger, ou qui entraîne une invalidité ou une incapacité, ou qui provoque une hospitalisation ou une prolongation d'hospitalisation.

Doivent également être transmises toutes les informations relatives à des faits nouveaux concernant le déroulement de la recherche, lorsque ce fait nouveau est susceptible de porter atteinte à la sécurité des personnes qui se prêtent à la recherche.

8 Statistiques

8.1 Calcul du nombre de sujets à inclure

Le nombre de sujets à inclure dans l'étude a été calculé en prenant en compte la variabilité du critère principal d’évaluation, à savoir la réactivité microcirculatoire en réponse

à l’action de l’acétylcholine ou du nitroprussiate. Nous nous sommes appuyé, pour estimer cette variabilité, sur plusieurs travaux publiés et quelques résultats personnels qui provenaient tous de protocoles légèrement différents de celui que nous avons retenus pour cette étude. En considérant qu’il s’agit d’un essai croisé dans lequel chaque sujet est son propre témoin, en comparaison bilatérale, avec un risque α fixé à 5% et un risque β fixé à 10% le nombre de sujets nécessaire serait au minimum de 22. Compte tenu qu’il est prévu d’utiliser un plan de randomisation basé sur un carré latin et que le nombre maximum de sujets qui peuvent être explorés chaque jour est de 3, l’effectif à inclure doit être un multiple de 3.

Nous avons donc prévu d’inclure au total 24 sujets qui seront répartis sur 8 groupes.

8.2 Description des méthodes statistiques utilisées pour l’analyse des données

Les effets de la consommation du jus d’orange sur la protection vasculaire et sur la fonction immunitaire seront analysés à l’aide d’un modèle ANOVA mixte, en faisant appel à la fonction PROC MIXED de SAS. Ce modèle statistique linéaire à effets mixtes permet d’observer la classification des données en fonction de diverses caractéristiques, grâce à des mesures répétées. Il est une extension directe du modèle linéaire à effet fixe et se base ainsi sur les mêmes hypothèses que les ANOVA, tout en considérant les données en tant qu’échantillon représentatif d’une population plus large que les données elles mêmes (effet aléatoire). Dans le cas présent, ce modèle comprendra comme effets fixes les facteurs durée (2 modalités), type de traitement (3 modalités), temps de mesure (4 modalités pour les paramètres sanguins, 3 modalités pour la réactivité vasculaire) et comme effets aléatoires la répartition des sujets dans les traitements. Il permettra d’évaluer l’effet des facteurs seuls et les éventuelles interactions pouvant exister entre eux. Dans le cas d’un effet significatif des facteurs, les différentes modalités seront comparées deux à deux.

On utilisera le coefficient de corrélation, la régression simple et la régression multiple type MaxR (maximum R^2) pour déterminer les relations entre les variables. Une étude exploratoire multidimensionnelle des données sera également envisagée en ayant recours à l’ACP (Analyse en Composantes Principales). Cette méthode factorielle permet d’étudier les relations entre les variables multiples de manière exploratoire, c'est-à-dire lorsque

l’exploitation des données n’est guidée par aucune hypothèse préalable. Par ailleurs l’ACP permet de positionner les individus, ou groupes d’individus, vis-à-vis de la structure révélée des variables.

9 Considérations pratiques

9.1 Déclarations officielles

Le protocole sera soumis au Comité de Protection des Personnes Sud Est 6.

En signant ce protocole, le promoteur et les investigateurs s’engagent à réaliser l’étude conformément aux recommandations de la déclaration d’Helsinki.

Parallèlement, le promoteur s’engage à envoyer une déclaration d’intention à la DGS avant le début de l’étude.

9.2 Contrôle et assurance de la qualité

L’investigateur s’engage au strict respect au cours du protocole expérimental, des règles de Bonnes Pratiques Cliniques et de la loi Huriet-Sérusclat du 20 décembre 1988.

L’investigateur se porte garant de l’authenticité des données recueillies dans le cadre de l’étude et accepte les dispositions légales autorisant le promoteur de l’étude à mettre en place un contrôle de la qualité.

9.3 Archivage des documents de l’étude

Les originaux des formulaires de consentement signés, la correspondance seront conservés par l’investigateur pendant une durée minimale de 15 ans, de même que le protocole et ses amendements, toute pièce administrative liée à l’étude, les données recueillies et le rapport d’analyse.

9.4 Contrat d’assurance

Paraphe du volontaire avec la mention « lu et compris »

Conformément aux dispositions de la loi Huriet-Sérusclat du 20 décembre 1988, le promoteur s'engage à contracter une assurance responsabilité civile.

9.5 Calendrier prévisionnel de l'étude

- Soumission du projet à l'avis du comité d'éthique et Obtention de l'approbation (M0)
- Recrutement des volontaires (de M 0 à M3)
- Réalisation de l'étude clinique (de M3 à M11 mois)
- Analyses biologiques, biochimiques et traitement statistique des données (M11 à M17)
- Rédaction de rapport et des publications scientifiques (M17 à M21)

9.6 Règles relatives à la publication

Ce projet est supposé avoir des répercussions très intéressantes en nutrition préventive.

Les résultats obtenus dans le cadre de cette étude seront soumis à la publication dans des revues internationales de nutrition de bons niveaux (type : Am. J. Clin. Nutr, J. Clin. Invest Journal of Nutrition, …), et feront l'objet de présentation dans des réunions scientifiques.

Paraphe du volontaire avec la mention « lu et compris »

Réferences

1.Ross, R. (1999) Atherosclerosis is an inflammatory disease. Am Heart J 138: S419-420.

2.Verma, S., Buchanan, M. R., & Anderson, T. J. (2003) Endothelial function testing as a biomarker of vascular disease. Circulation 108: 2054-2059.

3.Ness, A. R., & Powles, J. W. (1997) Fruit and vegetables, and cardiovascular disease: a review. Int J Epidemiol 26: 1-13.

4.Joshipura, K. J., Hu, F. B., Manson, J. E., Stampfer, M. J., Rimm, E. B., Speizer, F. E., Colditz, G., Ascherio, A., Rosner, B., Spiegelman, D., & Willett, W. C. (2001) The effect of fruit and vegetable intake on risk for coronary heart disease. Ann Intern Med 134: 1106-1114.

5.Bazzano, L. A., He, J., Ogden, L. G., Loria, C. M., Vupputuri, S., Myers, L., & Whelton, P. K. (2002) Fruit and vegetable intake and risk of cardiovascular disease in US adults: the first National Health and Nutrition Examination Survey Epidemiologic Follow-up Study. Am J Clin Nutr 76: 93-99.

6.Vivekananthan, D. P., Penn, M. S., Sapp, S. K., Hsu, A., & Topol, E. J. (2003) Use of antioxidant vitamins for the prevention of cardiovascular disease: meta-analysis of randomised trials. Lancet 361: 2017-2023.

7.Manach, C., Mazur, A., & Scalbert, A. (2005) Polyphenols and prevention of cardiovascular diseases. Curr Opin Lipidol 16: 77-84.

8.Arts, I. C., & Hollman, P. C. (2005) Polyphenols and disease risk in epidemiologic studies. Am J Clin Nutr 81: 317S-325S.

9.Manach, C., Williamson, G., Morand, C., Scalbert, A., & Remesy, C. (2005) Bioavailability and bioefficacy of polyphenols in humans. I. Review of 97 bioavailability studies. Am J Clin Nutr 81: 230S-242S.

10.de Koning, E. J., & Rabelink, T. J. (2002) Endothelial function in the post-prandial state. Atheroscler Suppl 3: 11-16.

11.Lefebvre, P. J., & Scheen, A. J. (1998) The postprandial state and risk of cardiovascular disease. Diabet Med 15: S63-68.

12.Nappo, F., Esposito, K., Cioffi, M., Giugliano, G., Molinari, A. M., Paolisso, G., Marfella, R., & Giugliano, D. (2002) Postprandial endothelial activation in healthy subjects and in type 2 diabetic patients: role of fat and carbohydrate meals. J Am Coll Cardiol 39: 1145-1150.

13.Brown, A. A., & Hu, F. B. (2001) Dietary modulation of endothelial function: implications for cardiovascular disease. Am J Clin Nutr 73: 673-686.

14.Middleton, E., Jr. (1998) Effect of plant flavonoids on immune and inflammatory cell function. Adv Exp Med Biol 439: 175-182.

15.Sanbongi, C., Suzuki, N., & Sakane, T. (1997) Polyphenols in chocolate, which have antioxidant activity, modulate immune functions in humans in vitro. Cell Immunol 177: 129-136.

16.Watzl, B., Bub, A., Briviba, K., & Rechkemmer, G. (2002) Acute intake of moderate amounts of red wine or alcohol has no effect on the immune system of healthy men. Eur J Nutr 41: 264-270.

Paraphe du volontaire avec la mention « lu et compris »

17. Bub, A., Watzl, B., Blockhaus, M., Briviba, K., Liegibel, U., Muller, H., Pool-Zobel, B. L., & Rechkemmer, G. (2003) Fruit juice consumption modulates antioxidative status, immune status and DNA damage. J Nutr Biochem 14: 90-98.
18. Chen, C. M., Li, S. C., Lin, Y. L., Hsu, C. Y., Shieh, M. J., & Liu, J. F. (2005) Consumption of purple sweet potato leaves modulates human immune response: T-lymphocyte functions, lytic activity of natural killer cell and antibody production. World J Gastroenterol 11: 5777-5781.
19. Hishikawa, K., Nakaki, T., & Fujita, T. (2005) Oral flavonoid supplementation attenuates atherosclerosis development in apolipoprotein E-deficient mice. Arterioscler Thromb Vasc Biol 25: 442-446.
20. Noguchi, N. (2002) Novel insights into the molecular mechanisms of the antiatherosclerotic properties of antioxidants: the alternatives to radical scavenging. Free Radic Biol Med 33: 1480-1489.
21. Vittal, R., Selvanayagam, Z. E., Sun, Y., Hong, J., Liu, F., Chin, K. V., & Yang, C. S. (2004) Gene expression changes induced by green tea polyphenol (-)-epigallocatechin-3-gallate in human bronchial epithelial 21BES cells analyzed by DNA microarray. Mol Cancer Ther 3: 1091-1099.
22. Vatanparast, H., Baxter-Jones, A., Faulkner, R. A., Bailey, D. A., & Whiting, S. J. (2005) Positive effects of vegetable and fruit consumption and calcium intake on bone mineral accrual in boys during growth from childhood to adolescence: the University of Saskatchewan Pediatric Bone Mineral Accrual Study. Am J Clin Nutr 82: 700-706.
23. He, F. J., & MacGregor, G. A. (2003) Potassium: more beneficial effects. Climacteric 3: 36-48.
24. Demigne, C., Sabboh, H., Remesy, C., & Meneton, P. (2004) Protective effects of high dietary potassium: nutritional and metabolic aspects. J Nutr 134: 2903-2906.
25. Sabboh, H., Horcajada, M. N., Coxam, V., Tressol, J. C., Besson, C., Remesy, C., & Demigne, C. (2005) Effect of potassium salts in rats adapted to an acidogenic high-sulfur amino acid diet. Br J Nutr 94: 192-197.
26. Leuzzi, U., Caristi, C., Panzera, V., & Licandro, G. (2000) Flavonoids in pigmented orange juice and second-pressure extracts. J Agric Food Chem 48: 5501-5506.
27. Manach, C., Scalbert, A., Morand, C., Remesy, C., & Jimenez, L. (2004) Polyphenols: food sources and bioavailability. Am J Clin Nutr 79: 727-747.
28. Jung, H. A., Jung, M. J., Kim, J. Y., Chung, H. Y., & Choi, J. S. (2003) Inhibitory activity of flavonoids from Prunus davidiana and other flavonoids on total ROS and hydroxyl radical generation. Arch Pharm Res 26: 809-815.
29. Franke, A. A., Cooney, R. V., Henning, S. M., & Custer, L. J. (2005) Bioavailability and antioxidant effects of orange juice components in humans. J Agric Food Chem 53: 5170-5178.
30. Trinchieri, A., Lizzano, R., Bernardini, P., Nicola, M., Pozzoni, F., Romano, A. L., Serrago, M. P., & Confalanieri, S. (2002) Effect of acute load of grapefruit juice on urinary excretion of citrate and urinary risk factors for renal stone formation. Dig Liver Dis 34: S160-163.
31. Tooke, J. E., Ostergren, J., & Fagrell, B. (1983) Synchronous assessment of human skin microcirculation by laser Doppler flowmetry and dynamic capillaroscopy. Int J Microcirc Clin Exp 2: 277-284.

Paraphe du volontaire avec la mention « lu et compris »

32. Hansell, J., Henareh, L., Agewall, S., & Norman, M. (2004) Non-invasive assessment of endothelial function - relation between vasodilatory responses in skin microcirculation and brachial artery. Clin Physiol Funct Imaging 24: 317-322.
33. Felmeden, D. C., & Lip, G. Y. (2005) Endothelial function and its assessment. Expert Opin Investig Drugs 14: 1319-1336.
34. Cominacini, L., Rigoni, A., Pasini, A. F., Garbin, U., Davoli, A., Campagnola, M., Pastorino, A. M., Lo Cascio, V., & Sawamura, T. (2001) The binding of oxidized low density lipoprotein (ox-LDL) to ox-LDL receptor-1 reduces the intracellular concentration of nitric oxide in endothelial cells through an increased production of superoxide. J Biol Chem 276: 13750-13755.
35. Dell'Agli, M., Busciala, A., & Bosisio, E. (2004) Vascular effects of wine polyphenols. Cardiovasc Res 63: 593-602.
36. Osiecki, H. (2004) The role of chronic inflammation in cardiovascular disease and its regulation by nutrients. Altern Med Rev 9: 32-53.
37. Ramsay, J. E., Ferrell, W. R., Greer, I. A., & Sattar, N. (2002) Factors critical to iontophoretic assessment of vascular reactivity: implications for clinical studies of endothelial dysfunction. J Cardiovasc Pharmacol 39: 9-17.
38. Ferrell, W. R., Ramsay, J. E., Brooks, N., Lockhart, J. C., Dickson, S., McNeece, G. M., Greer, I. A., & Sattar, N. (2002) Elimination of electrically induced iontophoretic artefacts: implications for non-invasive assessment of peripheral microvascular function. J Vasc Res 39: 447-455.
39. Rassaf, T., Feelisch, M., & Kelm, M. (2004) Circulating NO pool: assessment of nitrite and nitroso species in blood and tissues. Free Radic Biol Med 36: 413-422.
40. Mosmann, T. R., & Sad, S. (1996) The expanding universe of T-cell subsets: Th1, Th2 and more. Immunol Today 17: 138-146.
41. Romagnani, S. (2000) The role of lymphocytes in allergic disease. J Allergy Clin Immunol 105: 399-408.
42. Whiteside, T. L. (1994) Cytokines and cytokine measurements in a clinical laboratory. Clin Diagn Lab Immunol 1: 257-260.
43. Hurst, N. P. (1987) Molecular basis of activation and regulation of the phagocyte respiratory burst. Ann Rheum Dis 46: 265-272.
44. Shou, J., Bull, C. M., Li, L., Qian, H. R., Wei, T., Luo, S., Perkins, D., Solenberg, P. J., Tan, S. L., Chen, X. Y., Roehm, N. W., Wolos, J. A., & Onyia, J. E. (2006) Identification of blood biomarkers of rheumatoid arthritis by transcript profiling of peripheral blood mononuclear cells from the rat collagen-induced arthritis model. Arthritis Res Ther 8.
45. Connolly, P. H., Caiozzo, V. J., Zaldivar, F., Nemet, D., Larson, J., Hung, S. P., Heck, J. D., Hatfield, G. W., & Cooper, D. M. (2004) Effects of exercise on gene expression in human peripheral blood mononuclear cells. J Appl Physiol 97: 1461-1469.
46. Twine, N. C., Stover, J. A., Marshall, B., Dukart, G., Hidalgo, M., Stadler, W., Logan, T., Dutcher, J., Hudes, G., Dorner, A. J., Slonim, D. K., Trepicchio, W. L., & Burczynski, M. E. (2003) Disease-associated

Paraphe du volontaire avec la mention « lu et compris »

expression profiles in peripheral blood mononuclear cells from patients with advanced renal cell carcinoma. Cancer Res 63: 6069-6075.

47.Mao, T. K., Powell, J., Van de Water, J., Keen, C. L., Schmitz, H. H., Hammerstone, J. F., & Gershwin, M. E. (2000) The effect of cocoa procyanidins on the transcription and secretion of interleukin 1 beta in peripheral blood mononuclear cells. Life Sci 66: 1377-1386.

48.Van Erk, M.J., Blom W.A., van Ommen B., & Hendricks, H.F. (2006) High-protein and high-carbohydrate breakfasts differentially change the transcriptome of human blood cells. Am J Clin Nutr. 84:1233-41.

49. Schinkovitz, A., Dittrich, P., & Wasxher, T.C. (2001) Effects of a high-fat meal on resistance vessel reactivity and on indicators of oxidative stress in healthy volunteers. Clin Physiol. 21:404-410.

Paraphe du volontaire avec la mention « lu et compris »

Liste des annexes

Annexe 1: figure 1 - schéma général de l'étude

Annexe 2: figure 2 - Déroulement de la visite d'inclusion (V1) et des visites de début de période (V2, V4 et V6)

Annexe 3 : figure 3 - déroulement des visites de fin de période (V3, V5 et V7)

Annexe 4 : document d'information et de consentement

Annexe 5 : CV des investigateurs

Paraphe du volontaire avec la mention « lu et compris »

Annexe 1

Figure 1: Déroulement général de l'étude

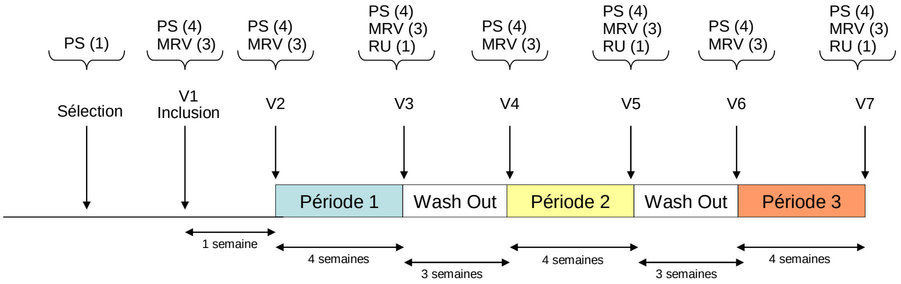

V : Visite au CIC
PS : Prise de sang
MRV : Mesure de Réactivité Vasculaire
RU : Recueil urinaire
(n) : Nombre de prélèvements ou de mesure

Durée totale : 19 semaines
Nombre de visite au CIC : 7 (+ 1 sélection)
Nombre de prise de sang : 29
Volume total de sang prélevé : 484 mL
Nombre de MRV : 21
Nombre de RU sur 24 heures : 3

Annexe 2 - Figure 2 :

Déroulement de la visite d'inclusion (V1) et des visites de début de période (V2, V4 et V6)

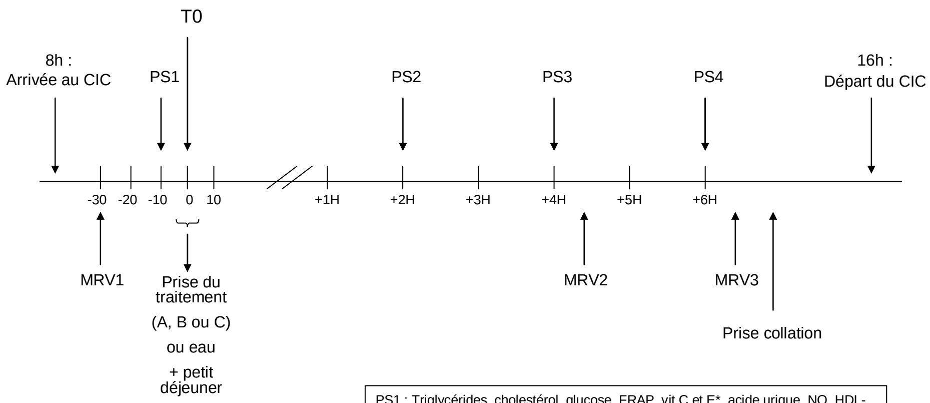

PS : Prise de sang

MRV : Mesure de réactivité vasculaire sur 20 minutes

PS1 : Triglycérides, cholestérol, glucose, FRAP, vit C et E*, acide urique, NO, HDL-cholestérol*, CRP*, IL-6*, vWF*, ICAM*, VCAM*, flavanones*, étude de la fonction immunitaire*

PS2 et 3 : Triglycérides, cholestérol, glucose, FRAP, vit C, acide urique, NO

PS4 : Triglycérides, cholestérol, glucose, FRAP, vit C, acide urique, NO, flavanones*

*Note : * indique les paramètres qui ne seront pas mesurés lors de la visite V1*

Paraphe du volontaire avec la mention « lu et compris »

Annexe 3

Figure 3 : Déroulement des visites de fin de période (V3, V5 et V7)

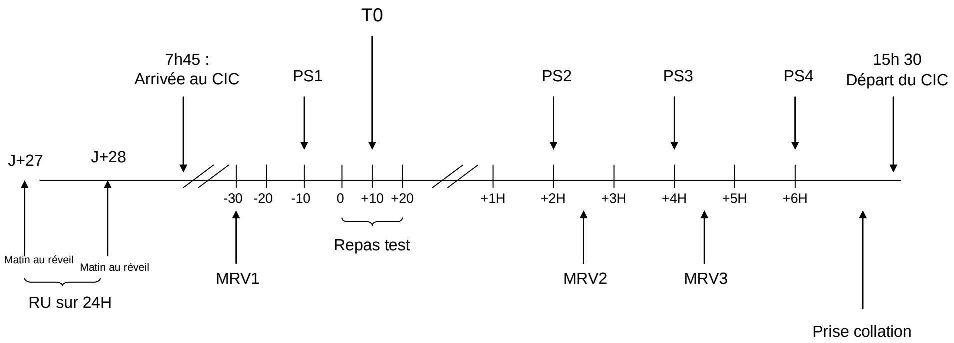

PS : Prise de sang

MRV : Mesure de réactivité vasculaire sur 20 min

RU : recueil des urines

PS1 : Triglycérides, cholestérol, glucose, FRAP, vit C et E, acide urique, NO, HDL-cholestérol, CRP, IL-6, vWF, ICAM, VCAM, étude de la fonction immunitaire, analyse transcritomique pour une moitié des sujets

PS2 : Triglycérides, cholestérol, glucose, NO, IL-6, vWF, ICAM-1, VCAM-1, analyse transcriptomique pour l'autre moitié des sujets

PS3 et 4 : Triglycérides, cholestérol, glucose, NO, IL-6, vWF, ICAM-1, VCAM-1

Paraphe du volontaire avec la mention « lu et compris »

Annexe 4 : DOCUMENT D'INFORMATION

remis à Mme, Melle, M. (nom, prénom) : ....
Adresse (complète) : ....
....
....
....

concernant l'étude suivante :

Effets de la consommation de jus d'orange sur la protection vasculaire et la fonction immunitaire : étude clinique sur la contribution spécifique des flavanones des agrumes

Le Docteur .... m'a proposé de participer à une recherche organisée par **le CHU de Clermont-Ferrand** sur **les effets de la consommation d'agrumes au niveau vasculaire et immunitaire** et m'a précisé que j'étais libre d'accepter ou de refuser cette proposition.

1) J'ai reçu et j'ai bien compris les informations suivantes :

- Objectif de l'étude :

Evaluer, chez des volontaires sains (hommes) d'âge mûr (50-65 ans) en léger surpoids et suivant leur régime alimentaire habituel, les effets à court et au bout d'un mois d'une consommation de jus d'orange ou de polyphénols d'orange purifiés sur les fonctions vasculaire et immunitaire.

Cette étude comporte 3 périodes expérimentales de 4 semaines chacune au cours desquelles les volontaires devront consommer quotidiennement l'un des 3 traitements suivants : A) 500ml de jus d'orange ; B) 500 ml de boisson sucrée + 1gélule placebo ; C) 500 ml de boisson sucrée + 1gélule de polyphénols d'orange. Chaque volontaire suivra les 3 traitements, les périodes correspondant à la prise de chaque traitement étant séparées par des intervalles de 3 semaines sans prise de traitement. La durée totale de votre participation à ce protocole est de 19 semaines au total.

- déroulement de l'étude :

Avant votre inclusion définitive, vous aurez un entretien avec un médecin et un examen médical de façon à vérifier que votre état de santé vous permet de participer sans risques particuliers pour votre santé à cette étude clinique. Le médecin investigateur vous expliquera en détail le déroulement de l'étude et répondra à toutes vos questions. Vous subirez un prélèvement de 13 mL de sang pour un bilan biologique initial et tous les résultats des analyses pourront vous être communiqué. Vous ne serez inclus définitivement dans le protocole que si ces examens sont normaux. Pendant toute la durée du protocole vous suivrez votre alimentation habituelle.

Sur toute la durée du protocole, vous passerez 7 journées au CIC : la première, une semaine avant le début de la première période, pour déterminer les valeurs basales (après consommation d'eau plate) des paramètres sanguins et de la réactivité vasculaire ; puis une journée en début et en fin de chaque période, ce qui représente 6 journées sur les 3 périodes expérimentales. Entre l'inclusion et la fin du protocole, il vous aura été fait 29 prises de sang, ce qui représentera un volume de sang prélevé de 484 ml, la mesure de réactivité vasculaire aura été faîte 21 fois, et 3 recueils urinaires sur 24h auront été effectués (1 à la fin de chaque période expérimentale).

Paraphe du volontaire avec la mention « lu et compris »

L'étude se déroulera sur 3 périodes de 28 jours avec pour chacune d'elles 2 journées passées au CIC, correspondant au début (J0) et à la fin de chaque période (J+28). Le premier jour de chaque période (J0) vous devrez arriver le matin à jeun au CIC pour consommer l'un des 3 traitements (jus d'orange ou boisson contrôle sucrée + gélule placebo ou boisson contrôle sucrée + gélule contenant des polyphénols d'orange) accompagné d'un petit déjeuner sans polyphénols. La prise de chacune de ces boissons sera précédée et suivie sur la journée de 4 prises de sang et de 3 mesures de la réactivité vasculaire sous-cutanée. Vous ne devrez rien consommer d'autre (sauf de l'eau) jusqu'à la fin des prélèvements et mesures prévue en milieu d'après midi. Avant de quitter le CIC, il vous sera servi une collation que vous consommerez sur place et ensuite vous pourrez rentrer chez vous. Entre J+1 et J+27, les doses quotidiennes (boisson et supplément) seront réparties en 2 prises équivalentes : la première au petit déjeuner et la seconde au moment du déjeuner. Entre J+27 (7h00) et J+28 (7h00) vous devrez recueillir vos urines de 24h dans un flacon (qui vous aura été préalablement remis à J0 lors de votre visite au CIC) et ce recueil sera déposé au CIC le matin de J+28 à votre arrivée au CIC pour la visite de fin de période. Lorsque vous arriverez à jeun au CIC le matin de J+28, il vous sera tout d'abord fait une mesure de réactivité vasculaire puis une prise de sang. Ensuite vous consommerez sur place un repas test riche en graisses, constitué de 1/3 de baguette, 30g de beurre et 180g d'emmental, apportant 1135 Kcal. La prise de ce repas sera accompagnée d'eau à volonté et il n'y aura pas de consommation du traitement à l'étude ce jour là. Le temps imparti pour la consommation du repas sera de 20 minutes. Vous resterez au CIC jusqu'à environ 16h30 et durant cette période, 3 autres prises de sang et 2 mesures de réactivité vasculaire seront réalisées. Vous ne devrez rien consommer d'autre (sauf de l'eau) jusqu'à la fin des prélèvements et mesures prévue en milieu d'après midi. Après la dernière prise de sang, vous prendrez sur place une collation et vous pourrez quitter le CIC.

- Contraintes liées à l'étude :

Pour que les résultats de cette étude soient utilisables, il est important que vous vous puissiez vous engager:

- à respecter les consignes pour la prise quotidienne des traitements (boisson et gélule)
- à respecter les consignes qui vous seront données de ne pas consommer (ou peu < 200 ml au total/j) de boissons riches en polyphénols (thé, café, vin, cacao, bière, lait de soja, jus de fruits) pendant toute la durée de l'étude.
- à vous rendre à 7 reprises, le matin à jeun, au Centre d'Investigation Clinique (CIC, Bât C, 58 rue Montalembert, 63003 Clermont-ferrand Cedex 1) pour consommer ce qui vous sera proposé (l'un des 3 traitements en début de période ou le repas test en fin de période) et à rester sur place jusqu'à la fin des prélèvements sanguins et des mesures de réactivité vasculaire prévue en milieu d'après midi.
- à informer le médecin de toute prise de médicament entre les visites.

Les inconvénients pouvant résulter des prélèvements sont d'éventuels malaises liés au stress de la piqûre, voire des problèmes infectieux ou inflammatoires. Ces dernières complications sont exceptionnelles dans la mesure où les précautions habituelles d'asepsie sont prises pour les éviter. Il n'existe aucun inconvénient prévisible pouvant résulter de la consommation de jus d'orange ou de polyphénols d'orange purifiés (équivalent à celui apporté par 500 ml de jus d'orange).ou de sucre aux doses prévues dans ce protocole d'étude qui correspondent à une apport nutritionnel.

Compte tenu des prélèvements de sang prévus pour les examens biologiques (qui correspondent grossièrement à un don du sang, toutefois étalé sur plus de 4 mois, il vous est demandé, par principe de ne pas procéder à un don de sang pendant 3 mois suivant la fin de votre participation à l'étude.

La durée totale de votre participation à l'étude est de 19 semaines.

Paraphe du volontaire avec la mention « lu et compris »

- Assurance liée à cette étude :

Comme l'exige la législation française en matière de recherches biomédicales, le promoteur a souscrit une assurance responsabilité civile contractée auprès de la compagnie SHAM N° de police………… , afin de pouvoir dédommager si votre état de santé s'altérait suite à votre participation à l'étude et dans la mesure où il pourra être établi que ces dommages sont la conséquence directe de l'étude.
Une attestation de cette assurance a été remise aux médecins en charge de votre suivi.

Les informations relatives à l'étude recueillies par l'investigateur sont traitées confidentiellement. En accord avec la Loi Informatique et Liberté, le nom des sujets est systématiquement remplacé par un N° de code dont la correspondance est connue des seuls médecins investigateurs.

J'accepte :

- que ces données puissent faire l'objet d'un traitement informatisé anonyme
- leur consultation par des collaborateurs participant à la recherche, désignés par le promoteur et éventuellement par un représentant des autorités de santé.
- mon inscription dans le Fichier National des personnes qui se prêtent à des recherches biomédicales sans bénéfice individuel direct (Art. L.209-17 Code de la Santé Publique).

2) Si je le souhaite, je pourrai à tout moment demander des informations complémentaires sur l'étude au personnel médical du Centre d'Investigation Clinique du CHU de Clermont-Fd au **04 73 17 84 10**. En cas d'urgence, et pendant toute la durée de l'étude, je pourrai joindre un des médecins en charge de l'étude en utilisant le système d'appel gratuit ALPHAPAGE. Pour cela composer le **08 36 60 10 10** qui vous met en contact avec une opératrice à laquelle vous donnerez le Code d'Appel **04 57 25** puis le N° de téléphone auquel le médecin peut vous rappeler
Dans le cas où mon état de santé serait altéré du fait de ma participation à l'étude, je serai en droit de recevoir des dédommagements dans le cadre d'un contrat d'assurance spécifique pris par le promoteur et conforme à la loi de Santé Publique du 9 Aout 2004.

3) Mon consentement, libre de toute pression, ne décharge pas les organisateurs de la recherche de leur responsabilité. Je conserve tous mes droits garantis par la loi. Si je le désire, je suis libre d'interrompre à tout moment ma participation à cette étude. Dans ces conditions, je m'engage à subir tous les examens médicaux nécessaires et à respecter toutes les instructions qui me seront données pour ma sécurité.

4) Je sais qu'en cours d'étude ma participation pourra être interrompue par les médecins investigateurs.

5) Toute information pertinente disponible au cours de l'étude et qui peut avoir des implications sur mon consentement pour la participation à l'étude me sera fournie par l'investigateur.

6) Je sais que cette étude a reçu l'approbation du CPP Sud Est 6 lors de sa séance du ……………….

7) Je recevrai une indemnité de 1130 euros lorsque la totalité de l'essai aura été réalisée. Je ne serais pas autorisé à participer à d'autres essais cliniques avant 3 mois après la fin de celui-ci.

Paraphe du volontaire avec la mention « lu et compris »

FORMULAIRE DE CONSENTEMENT

De M. ....(Nom, Prénom)

Né(e) le ....

Effets de la consommation de jus d'orange sur la protection vasculaire et la fonction immunitaire : étude clinique sur la contribution spécifique des flavonones des agrumes

Le Docteur .... m'a proposé de participer à l'étude organisée par **le CHU de Clermont-Ferrand.** Il m'a précisé que je suis libre d'accepter ou de refuser ; ceci n'affectera en rien nos relations. Les objectifs, les risques et la durée de cette étude et sa réalisation pratique m'ont été clairement expliqués. J'ai bien compris toutes les informations qui m'ont été fournies.

Je pourrai à tout moment demander une information complémentaire au médecin. Je recevrai pendant toute la durée de l'étude et quand je le désirerai, une information sur le déroulement de l'essai. Toute nouvelle information pertinente au cours de l'étude pouvant modifier mon consentement me sera fournie par l'investigateur.

Il m'a été signalé que les données me concernant pourraient faire l'objet d'une information partielle selon les modalités prévues par la loi, sans que mon identité ne soit révélée. J'accepte que ces résultats soient consultés par des personnes soumises au secret professionnel et collaborant à cette recherche et par des personnes habilitées par un médecin pour le compte du promoteur et/ou que ces résultats soient transmis aux Autorités Médicales légalement concernées en France et dans tout autre pays. J'accepte que les données enregistrées à l'occasion de cette recherche puissent faire l'objet d'un traitement informatisé par le promoteur ou pour son compte. J'ai bien noté que le droit d'accès prévu par la loi « Informatique et Liberté » (article 40) s'exerce à tout moment auprès du Docteur DUBRAY, médecin coordonnateur du Centre d'Investigation Clinique. Je pourrai exercer mon droit de rectification auprès de lui.

Mon consentement ne décharge pas les organisateurs de la recherche de leurs responsabilités. Je conserve tous mes droits garantis par la loi. Si je le désire, je serai libre à tout moment d'arrêter ma participation à cet essai. J'en informerai alors immédiatement un des médecins en charge de cette étude.

Je m'engage à ne cacher, aux médecins investigateurs, aucune information relative à mon état de santé et à mes habitudes de vies et à répondre en toute franchise aux questions qui me sont posées.

Je sais que cette étude a reçu l'approbation du CPP Sud Est 6 lors de sa séance du ……………….

J'ACCEPTE DE PARTICIPER A CETTE RECHERCHE DANS LES CONDITIONS PRECISEES

DANS LE DOCUMENT D'INFORMATION QUI M'A ÉTÉ REMIS AVEC CE FORMULAIRE ET

QUE J'AI LU AVEC ATTENTION.

Fait à ...., le ....

Signature du sujet : **Signature du médecin investigateur :**
(précédée de la mention « Lu et compris »)

Paraphe du volontaire avec la mention « lu et compris »

Annexe 5 : CV des investigateurs

CURRICULUM VITAE

Gisèle PICKERING
Née le 30 mai 1958 à Chamalières (Puy-de-Dôme)

N° d'inscription à l'ordre : 63/4083
N° ADELI : 63 10 4083 9

TITRES UNIVERSITAIRES

Doctorat d'Etat de Docteur en Pharmacie – 1984
Doctorat de Médecine – 1990
Doctorat d'Université – 1997
Capacité de Médecine et Biologie du Sport – 1997
Diplôme d'Etudes Supérieures Spécialisées en Administration des entreprises – 1983

FONCTIONS UNIVERSITAIRES

Assistante universitaire –Université de Cork (Irlande)
Assistante des Universités – 1999-2004
Maître de Conférences des Universités depuis 2004

FONCTIONS HOSPITALIERES

Médecin Attachée des Hôpitaux -1996-1999
Assistante des Hôpitaux – 1999-2004
Praticien hospitalier depuis 2004 Service de Pharmacologie Clinique

AUTRES FONCTIONS

Responsable du Groupe de travail spécifique Douleur et sujet âgé Société Française d'Evaluation et Traitement de la Douleur (SETD) depuis 2003
Consultante à la direction Régionale Jeunesse et Sports Auvergne – 1997-1998

SOCIETES SAVANTES

Membre de la Société Française de Pharmacologie
Membre de l'Association pour le Développement de la Pharmacologie Clinique
Membre de la Société Française de Gérontologie et Gériatrie
Membre de la Société Française d'Evaluation et Traitement de la Douleur (SETD)
Membre de l'International Association for the Study of Pain (IASP)

Paraphe du volontaire avec la mention « lu et compris »

CURRICULUM VITAE

Claude DUBRAY
Né le 20 juin 1951 à Chamalières (Puy de Dôme)

N° d'inscription à l'ordre : 63 / 4062
N° ADELI : 63 10 4062 3

TITRES UNIVERSITAIRES

- Doctorat d'Etat en Médecine - 1982
- Certificat d'Etudes Spéciales de Pédiatrie et de Puériculture - 1982
- Diplôme d'Etudes et de Recherches en Biologie Humaine (DERBH) - 1985
- Doctorat d'Université (Décret 1984) en Pharmacologie - 1992.

FONCTIONS UNIVERSITAIRES - Faculté de Médecine de Clermont Ferrand

- Chef de Clinique des Universités - 1983-1984
- Maître de Conférences des Universités - 1993-1997
- Professeur des Universités depuis septembre 1997

FONCTIONS HOSPITALIERES - C.H.U. de Clermont Ferrand

- Interne des Hôpitaux de 1978 à 1981
- Médecin Attaché des Hôpitaux de 1982 à 1983
- Assistant des Hôpitaux de 1983 à 1984
- Praticien Hospitalier depuis 1993 Service de Pharmacologie Clinique et Toxicologie
- Responsable du Centre de Pharmacologie Clinique / Centre de Recherche Clinique du CHU de Clermont-Ferrand

AUTRES FONCTIONS

I.N.S.E.R.M.

- Chargé de Recherches au titre des postes d'accueil INSERM dans l'Unité U 195 - 1982-1983

INDUSTRIE PHARMACEUTIQUE (Centre de Rech. des Laboratoires SANDOZ France)

- Responsable du Service de Pharmacologie Clin. et Exp. - 1984-1991
- Directeur Adjoint du Centre de Recherches - SANDOZ France - 1989-1993
- Responsable du Département de Pharmacologie Humaine (regroupant les services de Pharmacologie Clinique, Pharmacologie Expérimentale et Pharmacocinétique) 1991-1993

DELEGUE REGIONAL A LA RECHERCHE CLINIQUE Région Auvergne, depuis Octobre 1999

SOCIETES SAVANTES

- Membre de la Société Française de Pharmacologie
- Membre de l'Association pour le Développement de la Pharmacologie Clinique
- Membre de l'International Association for the Study of Pain (IASP)
- Membre de la Société D'étude et de Traitement de la Douleur (SETD) (Trésorier 1995-2001
- Membre de la Société des Neurosciences
- Membre de l'Association pour la Recherche en Physiologie de l'Environnement
- Membre de la Société Internationale pour le Développement des Recherches sur le Magnésium

Paraphe du volontaire avec la mention « lu et compris »

DUALE Christian, Jean

Né le 11 Décembre 1964 à Conches (Eure, France)
Lié par un Pacte Civil de Solidarité, deux enfants
Nationalité française
Service National effectué (1992)

N° inscription à l'Ordre des Médecins : 63 / 3897
N° ADELI : 63 10 3897 3

Adresse professionnelle :
Centre d' Investigation Clinique 501
Bâtiment 3C - CHU de Clermont-Ferrand
BP69
63003 Clermont-Ferrand Cedex 1
Téléphone professionnel : 04 73 17 84 10
e-mail : cduale@chu-clermontferrand.fr

Situation professionnelle :
Médecin des Hôpitaux, Anesthésiste Réanimateur, Praticien Hospitalier plein temps, Médecin Délégué au Centre d'Investigation Clinique 501 (Pr Claude DUBRAY, C.H.U. de Clermont-Ferrand).

Langues parlées : anglais, italien, portugais

Titres Universitaires :
Baccalauréat (série C), Académie de Clermont-Ferrand (1981)
Etudes de Médecine, UFR de Clermont- Ferrand (1981 – 1987)
Internat en Médecine, UFR de Clermont- Ferrand (1987)
Docteur en Médecine (DES d'Anesthésie et Réanimation Chirurgicale), UFR Médecine Clermont- Ferrand (1992)
Chef de Clinique des Universités, UFR de Clermont- Ferrand, 1992
Maîtrise de Sciences Biologiques et Médicales, UFR Médecine Clermont- Ferrand (1993)
Médecin des Armées (réserviste) (1994)
Diplôme d'Etudes Approfondies de Neurosciences, Université de Paris VI (1995)
Docteur de l'Université d'Auvergne (Spécialité Neurosciences), Clermont-Ferrand (2000)
Habilitation à Diriger les Recherches (Spécialité Anesthésiologie – Réanimation Chirurgicale), Université d'Auvergne (Faculté de Médecine de Clermont-Ferrand) (2005)

Titres et Fonctions Hospitalières
Interne des Hôpitaux, CHU de Clermont- Ferrand (1987)
Médecin Aspirant, Service de Santé des Armées, CHA Dominique Larrey (Versailles) et CHA Baudens (Bourges) (1991)
Médecin Attaché, CHG de Bourges (1992)
Assistant des Hôpitaux, CHU de Clermont- Ferrand (1992)
Praticien Hospitalier à titre provisoire, CHU de Clermont- Ferrand (1995)
Praticien Hospitalier, CHU de Clermont- Ferrand (1996)
Médecin Délégué au CIC 501 (Pr DUBRAY), CHU de Clermont- Ferrand (2006)
Membre de la Délégation à la Recherche Clinique, CHU de Clermont-Ferrand (2004-2006)

Paraphe du volontaire avec la mention « lu et compris »
