# Supplementary material for: Hesperidin Displays Relevant Role in the Nutrigenomic Effect of Orange Juice on Blood Leukocytes in Human Volunteers: A Randomized Controlled Cross-Over Study
Source: PLoS One. 2011 Nov 16;6(11):e26669. doi: 10.1371/journal.pone.0026669 (PMC3217928; doi:10.1371/journal.pone.0026669)
Supplement: Table S1 — Main constituents and phytochemicals contents in the test drinks (500 ml) used in the study. (DOC) [file pone.0026669.s003.doc]

**SUPPLEMENTAL TABLE S1**

|  |  |  | |  | |
| --- | --- | --- | --- | --- | --- |
| Main constituents and phytochemicals contents in the test | | | |  | |
| drinks (500 ml) used in the study | |  | |  | |
|  | Orange Juice | Control Drink | |  | |
|  |  |  | |  | |
| Total Carbohydrates (g) | 45 | 45 | |  | |
| Total Organic Acids (g) | 4.72 |  | |  | |
| Total Minerals (g) | 1.31 |  | |  | |
| Total Pectins (mg) | 568 |  | |  | |
|  |  |  | |  | |
| Total Vitamin C (mg) | 180 |  | |  | |
| a-tocopherol (mg) | 1.05 |  | |  | |
| Vitamin B9 (mg) | 0.085 |  | |  | |
| Total Carotenoids (mg) | 0.135 |  | |  | |
| Flavonoids (mg) | 341.9 |  | |  | |
| *Hesperidin* | *292* |  | |  | |
| *Narirutin* | *47.5* |  | |  | |
| o*thers* | 2.4 |  | |  | |
|  |  |  | |  | |
| Energy (Kcal) | 194 | 180 | |  | |
|  |  |  | |  | |
| Data are mean values |  |  | |  | |
| Carbohydrates include: sucrose (50%), glucose (25%), fructose (25%) | | | | | |
| Minerals include: Ca,Mg, Cu, Fe, Na, P, K, Zn | | |  | |  |
| Organic acids include: citric acid, isocitric acid and malic acid | | | | |  |
| Total carotenoids include: b-caroten, b-cryptoxanthin and lutein | | | | |  |
| Others flavonoids quantified include sinensitin, nobiletin and tangeretin | | | | | |
